# From Trauma to Innovation: A Comparative History of Limb Amputation and Prosthetics in Humans and Ungulates from Historical and Zooarchaeological Perspectives

**DOI:** 10.3390/ani16203278

**Published:** 2026-10-17

**Authors:** Jędrzej Siuta, Ernest Plichta, Edyta Pasicka

**Affiliations:** 1 Department of Forensic Medicine, Wroclaw Medical University, Mikulicza-Radeckiego 4, 50-345 Wrocław, Poland; jedrzej.siuta@umw.edu.pl; 2 Department of Biostructure and Animal Physiology, Faculty of Veterinary Medicine, Wrocław University of Environmental and Life Sciences, Kożuchowska 1/3, 51-631 Wrocław, Poland; 126535@student.upwr.edu.pl

**Keywords:** limb amputation, veterinary history, zooarchaeology, paleopathology, equine orthopedics, historical prosthetics, animal welfare, comparative surgery

## Abstract

Amputation and prosthetics represent vital themes in the history of medicine and zooarchaeology, reflecting the boundaries of therapeutic capabilities and evolving human–animal relationships. This study provides a comprehensive, comparative historical analysis of limb amputation procedures, surgical tools, haemostasis, and prosthetic rehabilitation in both humans and ungulates. A narrative literature review was conducted across electronic databases (PubMed, Web of Science, Scopus, Google Scholar, and the Cochrane Library) and digital repositories to evaluate archaeological evidence, ancient legal frameworks, and medieval/early modern medical and veterinary treatises. The historical evolution reveals striking cross-disciplinary technical analogies. Early modern surgical instruments (amputation knives and saws) and basic haemostatic approaches (fire cauterization transitioning to vascular ligation) developed along parallel trajectories in human and veterinary surgeries. However, the post-operative application of prosthetics reveals sharp biomechanical contrasts. While human prosthetics primarily addressed locomotion and cosmetics, veterinary prosthetics in heavy ungulates (horses and cattle) faced critical biological barriers, most notably contralateral limb overload leading to laminitis. Conversely, modern cases in lighter species (donkeys, alpacas, and sheep) demonstrate significantly higher adaptive success. Integrating human medical milestones with veterinary history provides a holistic framework for understanding paleopathologies and the cultural shift from culling injured livestock toward advanced, empathetic veterinary bioengineering.

## Simple Summary

This study was undertaken to understand how surgical techniques and human attitudes toward limb removal and artificial limbs have changed over time in both human and veterinary medicine. While amputation has long been a life-saving procedure in human medicine, it has historically represented a catastrophic, often fatal event for large hooved animals. Due to their heavy body mass, animals like horses and cattle suffer from severe complications when they lose a leg, most notably the dangerous overloading of the remaining healthy limbs. By examining ancient texts, historical veterinary treatises, and modern clinical records, this paper traces the evolution of surgical tools, blood loss control, and pain management. It highlights how cultural shifts and modern bioengineering, such as specialized artificial limbs that attach directly to the bone, are reshaping contemporary veterinary care. Our work achieves its aim by demonstrating that while body size remains a major challenge, animals have successfully transitioned from being treated as purely economic assets to patients deserving of advanced, empathetic medical care.

## 1. Introduction

When discussing amputation procedures, several distinct aspects must be addressed: general historical accounts with a timeline of primary historical sources; the types of instruments utilized for amputation; operative techniques regarding the method of limb removal; methods of haemostasis; the application of anaesthesia; and post-operative prosthetics. The issue of asepsis and antisepsis (which became widespread enabling factors for viable surgical and prosthetic outcomes in the second half of the 19th century) will not be discussed in detail in this study, as it represents a general medical milestone not specific to amputation. Nonetheless, their institutionalization marks the baseline from which modern, complication-free bone resections became possible. Because the field of amputation is extraordinarily vast (entire volumes have been dedicated to specific aspects mentioned), and this paper represents a comparative review of the history of amputation and prosthetics in humans and animals (primarily ungulates), it provides the reader with only a general outline of these issues, focusing chiefly on limb amputation without delving into specialized details, which interested readers can find in the cited literature.

Despite the expanding volume of literature on independent human and veterinary surgical histories, there remains a critical knowledge gap regarding the shared evolutionary trajectory of limb amputation and prosthetic development across these two domains. Specifically, a unified synthesis evaluating how historical human surgical milestones influenced veterinary practices—and how the unique biomechanical constraints of heavy quadrupeds limited the adoption of these techniques—has not yet been compiled. To address this gap, this narrative review is guided by three primary research questions: (1) How did historical shifts in surgical instrumentation and vascular control transfer from human to veterinary medicine? (2) What specific anatomical and physiological boundaries historically limited prosthetic success in heavy ungulates compared to humans? and (3) How have modern bioengineering and shifting animal welfare paradigms redefined amputation from an economic death sentence to a viable therapeutic option?

## 2. Materials and Methods

To provide a purposeful and structured narrative overview rather than a systematic registration of cases, this study synthesizes evidence from key historical, zooarchaeological, veterinary, and human medical sources. The methodology focused on synthesizing evidence from historical, osteological, veterinary, and human medical sources published up to August 2026. The literature search cut-off was set to August 2026 to encompass all peer-reviewed database entries up to the final manuscript revision cycle. Primary historical sources and treatises were purposefully selected based on their historical authority, technological representation of specific eras, and verified presence within major international digital repositories.

### 2.1. Search Strategy and Information Sources

The literature search was performed across major electronic databases, including PubMed, Web of Science, Scopus, Google Scholar, and the Cochrane Library. To capture relevant historical and zooarchaeological data, specialized repositories such as the Wellcome Collection, the U.S. National Library of Medicine Digital Collections, and the Bibliothèque nationale de France (BnF) manuscript archives were manually searched.

The search strategy utilized combinations of the following Medical Subject Headings (MeSH) terms and keywords:

Medical/Veterinary terms: “amputation”, “prosthesis”, “prosthetics”, “orthopedics”, “hemostasis”, “cauterization”, “anesthesia”, “laminitis”, “contralateral limb overload”.

Species-specific terms: “human”, “ungulate”, “equine”, “horse”, “bovine”, “cattle”, “small ruminant”, “goat”, “sheep”, “alpaca”.

Historical/Archaeological terms: “history of medicine”, “history of veterinary medicine”, “zooarchaeology”, “paleopathology”, “medieval medicine”, “Mamluk”, “ancient Rome”, “prehistoric”.

### 2.2. Inclusion and Exclusion Criteria

Studies, treatises, and archaeological reports were included if they met the following criteria:

- Discussed the surgical techniques, instrumentation, or outcomes of limb amputation in either humans or ungulates;
- Provided historical, osteological, or zooarchaeological evidence of survival following severe limb trauma, traumatic joint lesions, or natural cortical remodeling;
- Evaluated the historical evolution of perioperative care (hemostasis and anesthesia) relevant to amputation.

Publications were excluded if they focused strictly on small domestic animals (e.g., canines and felines), lacked historical or comparative relevance, or provided anecdotal evidence without reproducible source citations or peer-reviewed osteological verification. No geographical restrictions were applied. Documents written in English, Polish, Latin, French, and translated historical texts from Arabic and Greek were evaluated.

### 2.3. Data Extraction and Synthesis

A chronological and thematic synthesis approach was employed. Extracted evidence was organized according to the structure of the review: (a) historical development of amputation in humans and ungulates; (b) surgical instruments; (c) operative techniques and amputation levels; (d) haemostatic methods and cauterization; (e) anaesthesia; and (f) prosthetic management, long-term complications, and cross-species biomechanical constraints. Particular attention was given to differences in body mass, amputation level, prosthetic strategy, complications, and long-term outcomes in reported animal cases. Human and veterinary evidence were subsequently compared to identify historical technological links and the anatomical and biomechanical factors that limited the transfer of human amputation and prosthetic techniques to large ungulates.

### 2.4. Limitations

Several limitations should be considered when interpreting the findings of this review. First, the available evidence is subject to publication bias, as unusual cases and successful outcomes may be more likely to be reported than unsuccessful or fatal cases. This applies to both human and veterinary literature and may result in an overestimation of survival and functional outcomes following limb amputation.

Second, historical sources are particularly susceptible to survival and preservation bias. Cases involving prolonged survival or outcomes considered noteworthy were more likely to be documented and preserved, whereas fatal or poorly documented cases may have been lost from the historical record. Language bias and uneven access to historical sources may have further limited the identification of relevant cases. Therefore, the absence of a documented case or zooarchaeological specimen of a surviving individual should not be definitively interpreted as evidence that such events did not occur, but rather highlights the boundaries of the archaeological preservation layer.

Finally, comparisons between human and animal cases should be interpreted cautiously. The two bodies of literature differ in reporting practices, historical context, follow-up, and assessment of functional outcomes, while anatomical, biomechanical, and management differences further limit direct extrapolation. The comparisons presented here should therefore be understood as identifying historical and biological parallels rather than establishing direct equivalence between human and animal outcomes.

## 3. General Historical Overview of Amputation in Humans and Animals

Limb amputations represent some of the oldest medical (and surgical) interventions known to humanity. In the case of the oldest Neolithic human skeletal remains exhibiting clear signs of limb removal, it is not always possible to definitively determine whether these amputations were intentional medical treatments or the direct result of traumatic injuries [1]. Taking into account the evolutionary development of human knowledge and skills, the first deliberate removals of a limb or its fragment were most likely the result of observing traumatic changes in individuals who managed to survive such injuries. A similar origin can likely be attributed to the knowledge of managing haemorrhage (indissolubly linked to amputation), which appeared in primitive medicine worldwide from the earliest times. Previously, a left arm amputation performed around 4900–4700 BCE in present-day France was considered the oldest likely medical amputation in humans [2]. Currently, the earliest documented medical limb removal is a case from approximately 31,000 years ago in present-day Borneo, where a young individual underwent an amputation of the distal 1/3 of the left lower limb and subsequently survived the procedure for about 6–9 years [3]. These insights are derived from archaeological findings and osteological examinations of human remains.

**Figure 1:**
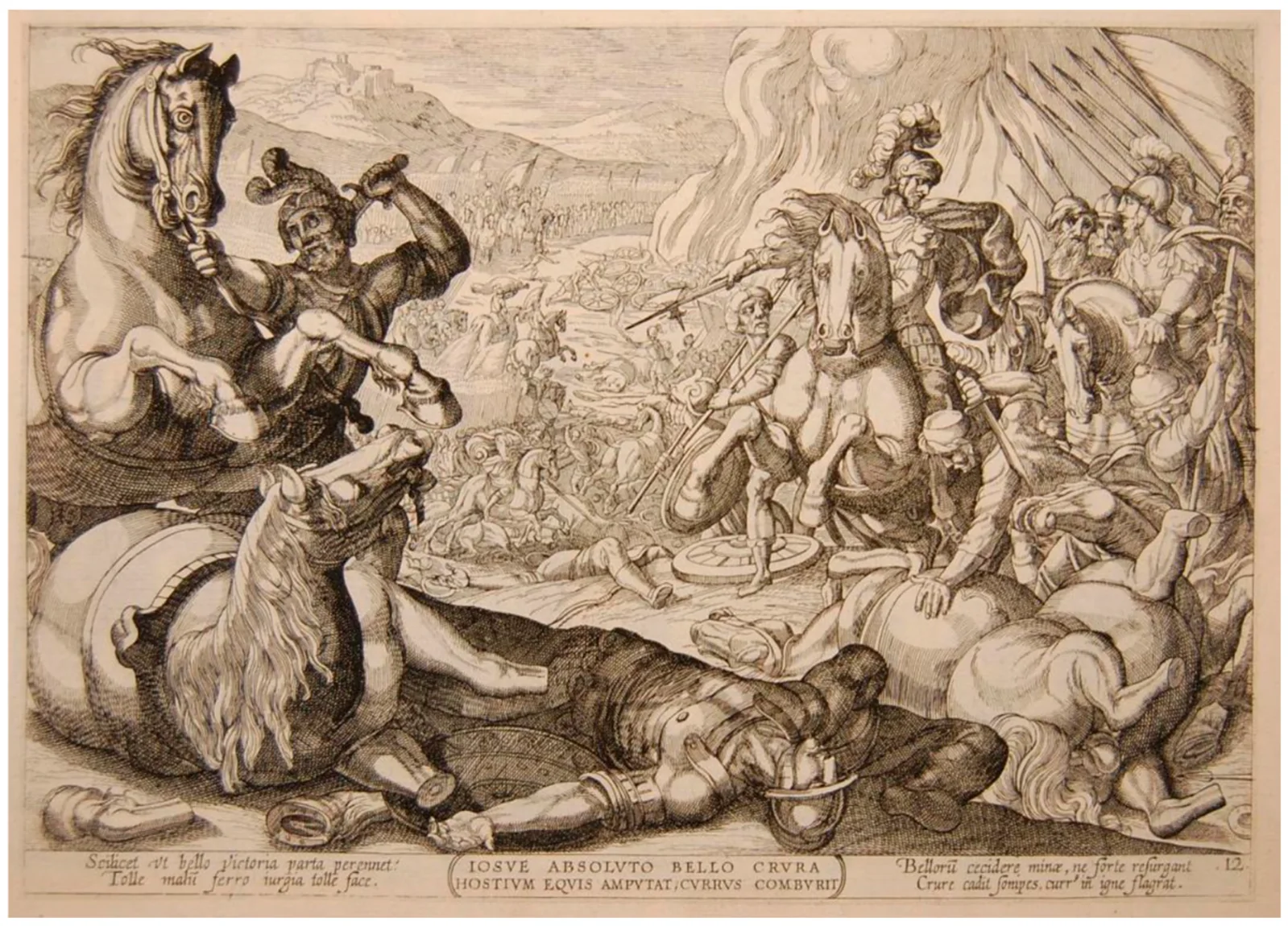
“*Iosue absoluto bello crura hostium equis amputat, currus comburit*” (Latin for: “Joshua, having ended the war, cuts the legs of the enemy’s horses and burns their chariots”)—an etching/engraving by Antonio Tempesta (late 16th/early 17th century) depicting a biblical scene from the Bronze Age [8]. The artwork illustrates the ancient military practice of intentional maiming of warhorses (hamstringing). It represents the early modern, purely mechanical understanding of the Latin term *amputat* (amputates/cuts off) as an act of destruction and permanent immobilization of the ungulate locomotor system, serving as a stark historical contrast to the subsequent development of therapeutic veterinary surgery and humane euthanasia. [Image courtesy of the British Museum/Wikimedia Commons (Public Domain); Museum number: BM_1948,0410.4.13] [7].

In stark contrast to human medicine, where amputation has served as a life-saving attempt for millennia, traditional veterinary medicine viewed severe orthopedic limb trauma in large animals, particularly horses, as a direct indication for euthanasia [4]. This approach stemmed from an exceptionally poor long-term prognosis in perissodactyl patients. Historically, human attitudes toward animals and their legal protection evolved across eras according to shifting utilitarian and economic needs [5]. An analysis of ancient legal frameworks, including paragraph 74 of the Hittite Laws, indicates that if a person broke the leg or horn of an ox, the perpetrator was obliged to purchase the disabled animal and provide the owner with a fully healthy individual or pay an equivalent of 2 silver shekels [6]. The complete absence of legal regulations regarding the return of animals after healing or completed convalescence permits the historical inference of a low efficacy of contemporary fracture treatments in large ruminants. In the realities of ancient military conflicts in the Near East, where horses and chariots constituted military power, the lack of effective orthopedic procedures resulted in the deep-rooted practice of intentional maiming of the locomotor systems of the enemy’s warhorses to permanently eliminate them from combat. This so-called hamstringing, consisting of the violent transection of the pastern tendons, was synonymous in the nomenclature and iconography of the era with a destructive, violent act of limb “removal” or “amputation,” completely devoid of any therapeutic or humane context, as illustrated in a late Renaissance engraving by Antonio Tempesta (Figure 1) [7].

A breakthrough in Near Eastern veterinary onomastic and clinical practice occurred through the work of the scholar Abu Bakr Al-Baytar, the court veterinarian in Mamluk Egypt during the 14th century [9]. In his writings, he implemented advanced protocols for constructing orthopedic splints, techniques for surgical access to bone structures, methods of soft-tissue cauterization, and individualized pharmacological protocols including the administration of immobilizing agents and analgesics for limb fractures in horses and cattle [9]. It is worth noting that the classical term for an animal doctor (*baytār*) derives directly from the Arabic root *batara*, defined in medieval dictionaries as incising, dissecting, or amputating [10]. Although Mamluk surgeons possessed theoretical knowledge regarding the removal of diseased structures, contemporary authors explicitly warned that errors by an inexperienced operator during incisions, cauterization, or amputation could cause permanent disability or death [10]. Despite this, Mamluk treatises from the 14th and 15th centuries, such as *Kitab al-Baytarah*, document the performance of other advanced invasive procedures, including dental operations (canine extractions in camels) and gynecological interventions involving the surgical removal of a dead fetus from a mare’s uterus [10]. While these advanced clinical protocols were flourishing in the Islamic world, the development of veterinary practice in medieval Europe followed a more decentralized path. In historical Poland, where the high economic value of large animals gradually transformed husbandry into a matter of state importance, traditional veterinary medicine relied for centuries on the activities of craftsmen and court personnel. As indicated by Janeczek et al., ref. [11] specialized falconers and dog handlers responsible for the health of their assigned animals operated at the Polish royal court as early as the 13th century. Yet, horses remained the primary focus of concern, and their treatment rested predominantly in the hands of blacksmiths and farriers. The first named Polish craftsman engaged in this profession was the blacksmith Jagusz from Wiślica, who treated the horses of King Władysław II Jagiełło at the turn of the 14th and 15th centuries. During the early modern period, equine medical care in Eastern Europe continued to be managed by these vernacular practitioners, widely referred to in regional historical records as farriers, marshals (*marscalci*), or horse leeches. Rather than executing invasive surgical resections, these practitioners focused almost exclusively on external topical applications, thermal cauterization, and bloodletting [11].

**Figure 2:**
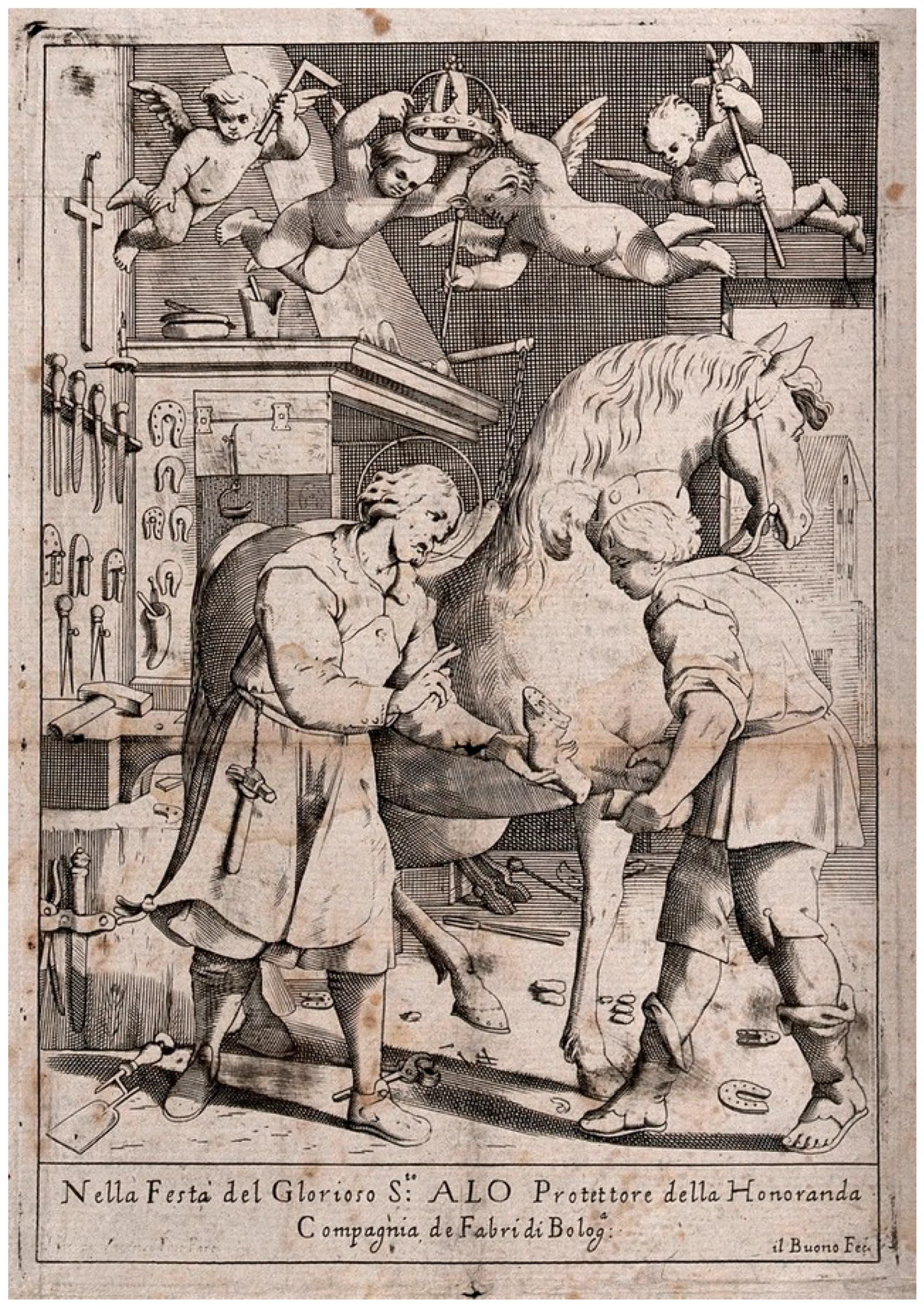
Saint Eligius executing the miraculous farriery procedure through temporary distal limb amputation and subsequent anatomical reattachment. Line engraving by “il Buono” (Floriano dal Buono, active 17th century), illustrating the intersection of spiritual hagiography, early podiatric craftsmanship, and the mechanical understanding of equine anatomy prior to the institutionalization of academic veterinary science. Source: Wellcome Collection (London, UK; Reference: 4830i) (Public Domain) [12].

This profound historical intersection of farriery, metallurgy, and early veterinary practice is conceptually embodied in the iconography of Saint Eligius (c. 588–660 CE), the patron saint of metalworkers, farriers, and veterinarians [12]. In late medieval and early modern European hagiographical narratives, a prominent miracle attributed to the saint involves the literal detachment (amputation) of a horse’s distal thoracic limb to facilitate precision shoeing on an anvil, followed by its successful anatomical reattachment without postoperative adverse effects (dal Buono, 1600–1699) (Figure 2) [12]. While strictly legendary and unscientific, this representation warrants historical analysis. It highlights a pre-modern cultural paradigm that conceptualized the equine locomotor apparatus through a purely mechanical lens, abstracting the biological barriers of soft-tissue ischemia, surgical shock, and vascular necrosis [12]. Furthermore, this miraculous narrative underscores a persistent historical desire for a fully reversible orthopedic intervention in equids—a striking historical contrast to the severe biological realities of contralateral limb overload and laminitis documented in contemporary veterinary bioengineering [4].

**Figure 3:**
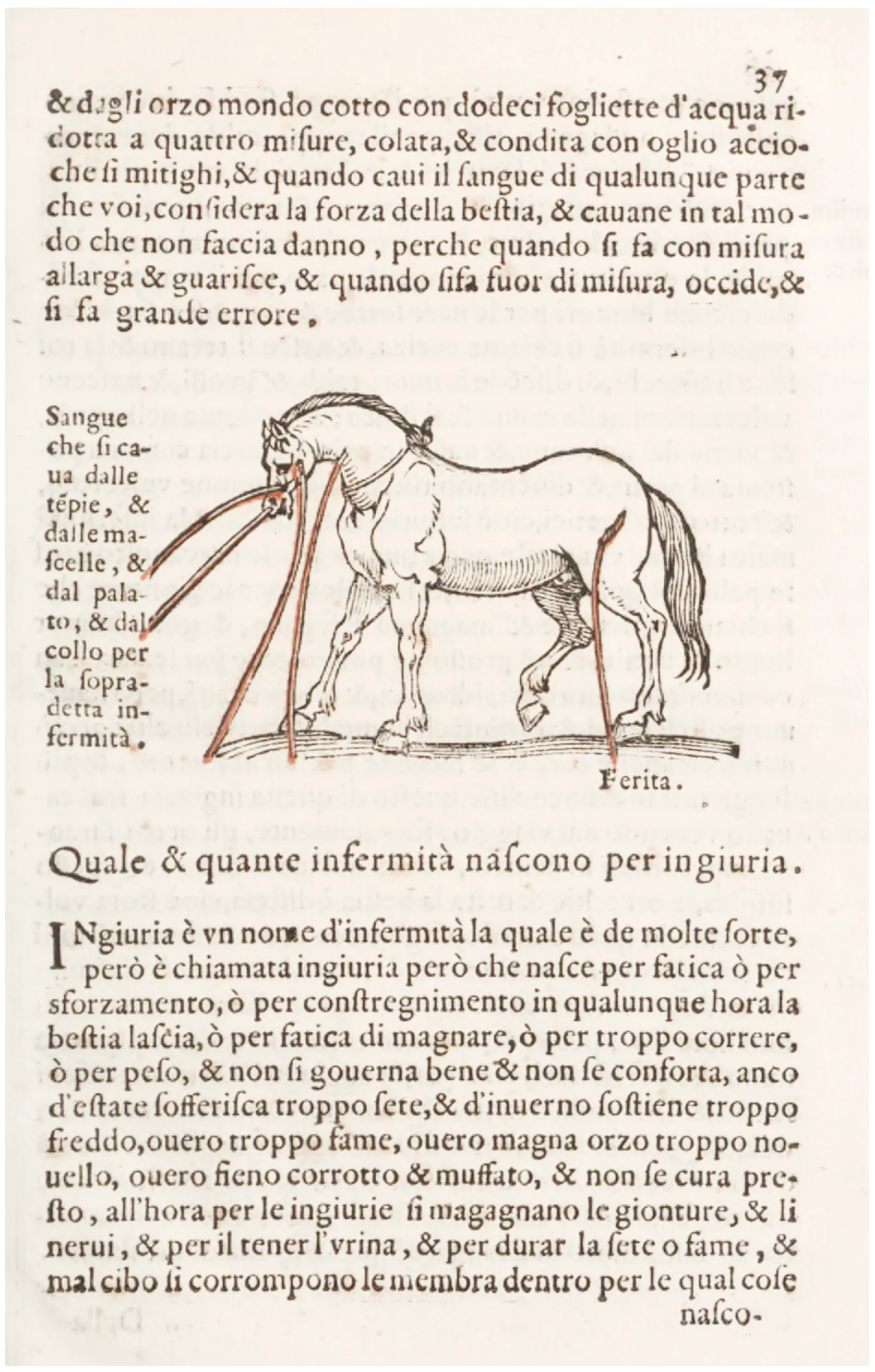
Schematic mapping of bloodletting (phlebotomy) points on the equine body. Woodcut from Filippo Scaccho’s “*Opera di Mescalzia*” (Treatise on Horse Medicine) (1591), illustrating early modern non-resectional methods aimed at reducing inflammation through humoral decompression. Source: National Library of Medicine (NLM) Digital Collections (Public Domain) [13].

Traditional hippiatry in the treatment of severe equine diseases was based exclusively on conservative and superficial methods. Evidence of this approach is the treatise by Filippo Scaccho, “*Opera di Mescalzia*” (1591) [13]. Early therapy was limited to bloodletting and deep cauterization of the limbs (Figure 3) in order to reduce inflammation, for example in the case of laminitis [13]. Renaissance practitioners consistently avoided radical surgical resections, such as amputation, even when dealing with advanced lameness or chronic orthopaedic conditions. This resulted from the fear of losing the anatomical continuity of the leg, a lack of knowledge about hoof biomechanics, and the inability to effectively offload the affected limb in such large animals [11].

Academic literature in the vernacular Polish language was born only in the 16th century [11,14,15]. A turning point was the 1532 publication of an anonymous work titled “*Sprawa a lekarstwa końskie*” (Horse Affairs and Medicines), which described symptoms and treatment methods for the most common equine diseases [16], opening access to veterinary knowledge for those unfamiliar with Latin. The same century brought further fundamental titles, such as “*Księgi o gospodarstwie*” (Books on Husbandry) from 1542—a translation of Pietro de’ Crescenzi’s work by Jędrzej Trzecieski [17] and “*Lekarstwa doświadczone*” (Experienced Medicines) by Marcin Siennik in 1564, which systematized contemporary surgical procedures, including bloodletting [18]. The primary native surgical instrument utilized for these common bloodlettings was a metal fleam, the oldest specimen of which on Polish lands (from an early medieval hillfort in Bródno) dates back to the end of the 11th century [15]. In the international Renaissance landscape, an important work was “*Opera di Mescalzia*” [13]. The author described seven primary equine diseases, including farcy (*farciminosa*), and introduced the modern principle of isolating sick individuals and deeply burying carcasses to prevent the spread of infections. These early modern measures established a historic baseline that eventually led to formalized military veterinary codes and field regulations regarding livestock management in early 20th-century Poland [19].

The fundamental development of invasive orthopedics in large animals occurred in the 20th century [20]. At the beginning of the last century, severe trauma to the locomotor apparatus in horses and cattle still constituted an absolute criterion for culling, and amputations were deemed ineffective; exceptions included tail docking and attempts to save valuable breeding stock or mares in late pregnancy [21]. The progressive mechanization of rural areas in the first half of the 20th century displaced the horse as a draft force, which initially—for economic reasons—resulted in the abandonment of complex surgical treatments [20]. However, this phenomenon led to a profound social transformation: horses acquired the status of companion and recreational animals, which birthed the contemporary approach to their welfare and provided an impulse for the development of life-saving techniques [20]. Consequently, contemporary advances in surgical techniques and biomedical engineering have led to partial limb amputation combined with subsequent prosthetics being considered a humane alternative to animal slaughter [4]. This procedure not only saves lives but, in selected cases, restores production and reproductive efficiency, as documented by clinical cases of mares and stallions that successfully continued their breeding careers after rehabilitation [22,23].

Concurrently, written sources regarding humans richly documented the development of this procedure as an established medical practice. The first description of a deliberate human amputation is recorded in the “Histories” (*Historiai*) of the famous Greek historian Herodotus (484–426 BCE) [24,25]. In the ninth book, he describes the account of a Persian soldier-seer, Hegesistratus, who, captured by the Spartans and bound in stocks, cut off a portion of his own foot to escape, subsequently replacing it with a wooden prosthesis after the wound healed to participate in further military campaigns [26]. Transitioning from historical narratives of self-inflicted trauma to formal clinical records, the earliest medical work containing a description of amputation as a standard surgical procedure is the treatise “On the Articulations” (*De Articulis*), part of the *Corpus Hippocraticum* attributed to Hippocrates [27,28], which describes the removal of the lower leg following post-traumatic ischemia and gangrene. In Greco-Roman times, limb amputations were detailed in the works of Aurelius Celsus (c. 53 BCE–c. 7 CE) and Galen (c. 130–c. 200) [25]. Mastectomies were described by the Byzantine surgeon Paul of Aegina (c. 625–690) in his work “Medical Compendium in Seven Books” [29].

During the Islamic Golden Age, a famous work describing medical amputations was the encyclopedia “*Kitab Al-Tasrif*” by the Arab physician Abulcasis (936–1013) [27,29,30]. Among later European medical texts, the 1367 work “*Chirurgia Magna*” by Guy de Chauliac—often called the “father of surgery”—must be noted for its descriptions of various limb amputation methods [31,32]. Although the Middle Ages did not generally abound in essential medical texts on surgical amputations, important works increased significantly from the 16th century onward [27,32,33,34]. The greatest advancement in human surgery occurred during the second half of the 19th and the first half of the 20th centuries, and medical knowledge in this field continues to develop today based on emerging biotechnologies.

From a zooarchaeological perspective, the macro-morphological analysis of faunal remains provides valuable baseline data on how ungulates historically adapted to severe limb trauma without veterinary intervention [35,36,37]. However, interpreting past veterinary care carries major limitations, as soft tissues rarely preserve, leaving bones as the sole analytical material [38]. Distinguishing natural biological processes from deliberate human healing is exceptionally difficult. Neatly aligned distal long-bone fractures with a longitudinal depression in the callus—traditionally attributed to splinting and bandaging in small livestock—have been identified identically in modern wild roe deer, where the indentation corresponds simply to the flexor tendons [39]. Spontaneous healing can occur via an “anatomical splint” (*attelle anatomique*) whenever an adjacent intact bone stabilizes the fracture [39]. Prophylactic and tragic human interactions are easier to detect, exemplified by a 14th-century calving dystocia at Téteghem (France) where a perimortem metacarpal fracture in a calf reflects a failed forced extraction [38]. Conversely, physical protection devices, such as Roman hipposandals used to secure pressure bandages on equids, provide objective evidence of orthopedic care [38,39].

Chronic pathologies regularly document the mechanical stresses of traction and riding. Osteoarthritis with extensive joint eburnation in cattle acetabula, femoral heads, metapodials, and vertebrae serves as a proxy for heavy draught labor [40]. To quantify these lesions systematically, a pathological index (PI/mPI) based on five changes of the first phalanx was developed using modern draught oxen [41,42]. Higher mPI values in hind limbs reflect severe dynamic loading during traction, charting the shift of cattle from multi-purpose wealth symbols to hard-worked trade commodities between the 5th and 14th centuries [42]. PI distribution also reflects site economy; highest indices (0.275) occurred at the Roman production villa of Torgny, outscoring urbanized vici like Liberchies or Namur, whereas late medieval sites like Koekelare show minimal lesions as horses assumed traction duties [41]. Furthermore, morphological analysis of pubic bones at Tiel-Passewaaij revealed that cows were routinely subjected to traction alongside oxen, mirroring classical accounts by Columella [40].

In equids, complex performance demands generated distinct skeletal responses, corroborated by recent extensive paleopathologic surveys of early medieval horses in Poland [43] and the Byzantine Theodosius Harbour at Yenikapı, Istanbul [44]. Limb lesions primarily involved distal segments; the high prevalence of splint bone exostoses and interosseous ligament ossification reflects intense duty at an early age [43,44]. Bone spavin (osteoarthritis of the tarsal joint) was similarly widespread [43,44]. The presence of advanced spavin with ankylosis indicates that horse keepers understood the disease course, intentionally maintaining lame animals until the painful acute phase subsided and mobility partially returned [43]. Conversely, thoracolumbar *spondylitis chronica deformans* and kissing spine syndrome were induced by riding stresses and ill-fitting saddles [43,44]. Brutal riding practices are evidenced at Yenikapı, where powerful curb-bits caused extensive inflammatory defects and perforations of the hard palate (*palatum durum*), concurrent with severe nuchal exostoses [44]. Direct evidence of mounted combat includes a lethal battle-axe/hammer cranial trauma at Ostrów Lednicki [43].

Crucial hoof disorders like laminitis (*pododermatitis aseptica diffusa*) have plagued horsemen for millennia, generating a rich historical record from Aristotle’s “barley disease” to Apsyrtus’s descriptions of gastrointestinal and road founder in the *Hippiatrika* compendium [45]. These texts align with paleopathological specimens from Yenikapı exhibiting *facies parietalis* osteolysis of the distal phalanx and advanced hoof cartilage ossification (sidebone) [44]. Historical treatment protocols targeting the “four humors” via severe phlebotomy, purgatives, or traumatic medieval wall resections eventually transitioned to modern rest-based regimens and corrective shoeing, such as heartbar shoes providing frog pressure [45].

The socioeconomic value of these large quadrupeds often determined their post-traumatic and post-mortem fates. Healed long-bone fractures in adult cattle and horses are exceptionally rare in ancient faunal assemblages because their low reduction success rate and poor functional prognosis prompted immediate economic culling [39]. Exceptions are almost exclusively restricted to juveniles under six months [39]. However, notable adult exceptions include a misaligned horse metacarpal from Iron Age Manching [39] and a completely consolidated metatarsal fracture with severe secondary tarsal ankylosis from Byzantine Yenikapı, proving that selected animals were actively nursed for at least six months post-injury [44]. Because no naturally healed cannon bones have been recorded in wild ungulates of comparable body mass, such rare domestic specimens confirm active human therapeutic intervention [39,44]. When survival was impossible, carcasses were thoroughly utilized; extensive cut and chop marks on early medieval horses from Poland, Téteghem, and Yenikapı demonstrate post-mortem skinning, butchery, or dietary consumption [38,43,44]. For exceptionally valued individuals, this economic lifecycle was replaced by emotional commemoration, as evidenced by the ritual deposition of draught cattle limbs at Ketton and the unique 13th–14th century English field names (e.g., *Thertheoxlaydede*) mapping the exact locations where prized working animals had died [42]. Furthermore, rare zooarchaeological specimens of ungulate appendicular skeletons demonstrating complete, naturally remodeled cortical rounding of distal bone shafts provide clear evidence of long-term survival following traumatic self-amputation or severe frostbite gangrene, long before modern orthopedic protocols were established.

These macro-morphological evidence and historical trajectories of both human and veterinary practices are comprehensively compared in Table 1.

## 4. Types of Instruments Utilized for Amputation and Related Procedures

Amputation is explicitly linked to the necessity of transecting tissues, therefore requiring sharp or pointed instruments. In ancient times, such tools most likely consisted of human fingernails, stone tools, and other items with sharp edges—fragments of rock, shells, plants, or animal teeth [33]. These implements were gradually replaced by tools made of copper, followed by bronze (from c. 3400 BCE), as well as tin, lead, and brass, ultimately yielding to iron objects [34]. Although the production of iron objects dates back to 1200 BCE, metal surgical instruments for tissue cutting were first described in the works of Hippocrates (c. 460–377 BCE), while steel spread for medical tool manufacturing only during Roman times [33,34].

**Figure 4:**
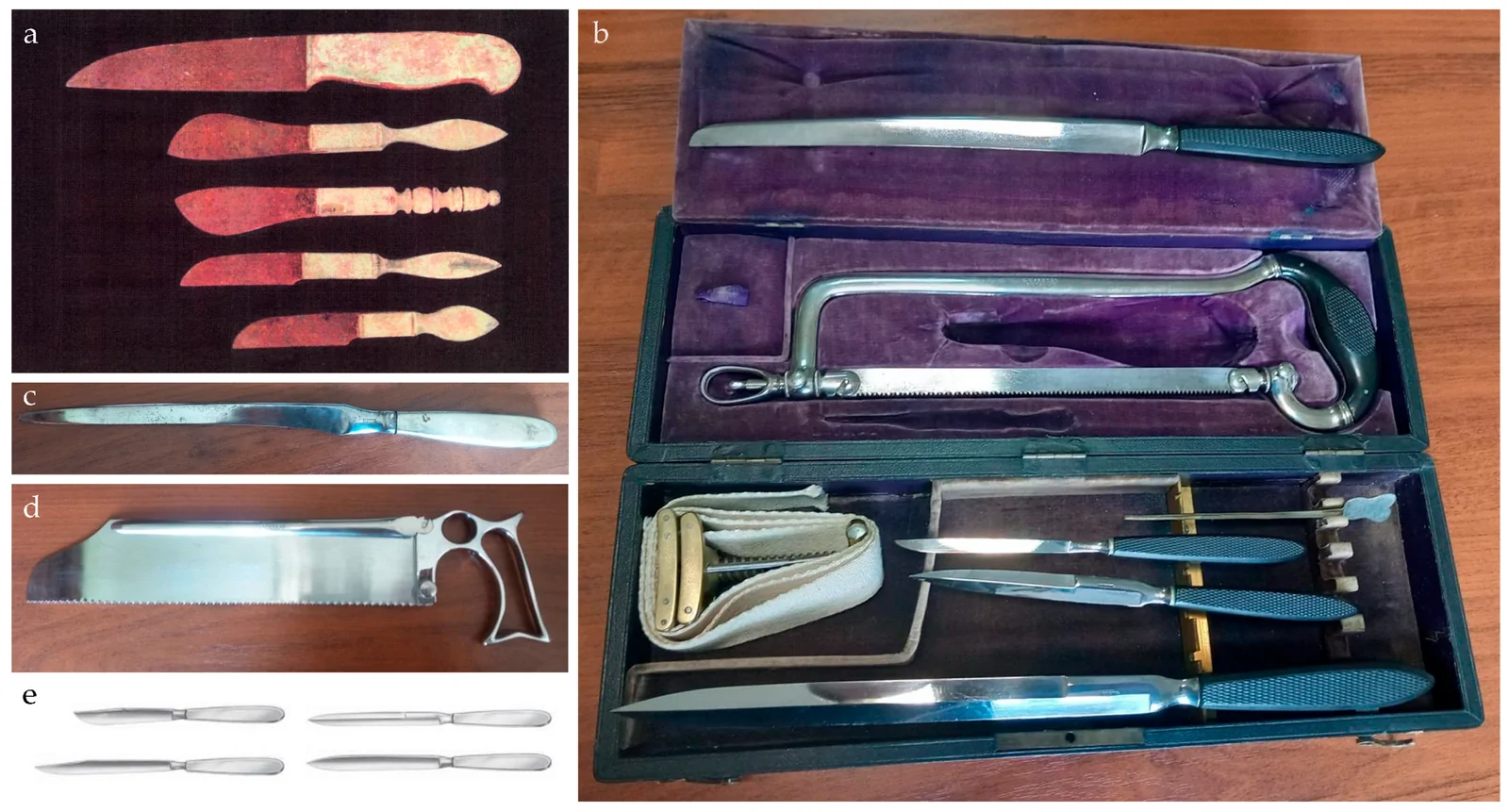
Historical evolution and modern examples of surgical cutting instruments and amputation sets: (**a**): scalpels from Roman times—iron blades 4.6 to 8 inches in length and bronze handles. From: Surgical Instruments from Ancient Rome, University of Virginia (online), [https://web.archive.org/web/20200221011435/http://exhibits.hsl.virginia.edu/antiqua/instruments/ (accessed on 9 August 2026), Public Domain] [47]; (**b**): amputation set, Haertel Breslau, 1880s; handles made of ebony. Private collection of Michał Wepsięć, photo: Jędrzej Siuta, reproducted with permission; (**c**): amputation knife, Pischel Breslau, 1920s, chrome plating steel. Collection of the Museum of Forensic Medicine, Wroclaw Medical University, photo: Jędrzej Siuta, reproduced with permission; (**d**): modern amputation saw (photo: J. Siuta, reproduced with permission); (**e**): modern amputation knives. From: Medimpent medical tools catalog (online); [https://www.medipment.pl/produkt/nopa-instruments-noze-amputacyjne-79276 (accessed on 9 August 2026), Public Domain] [48].

While historical artifacts exist in the form of metal surgical instruments from various eras, such as Roman surgical knives with iron blades and bronze handles [33,34,46] (Figure 4a), most surviving antique “amputation sets” date to the 18th and 19th centuries. Their handles were typically crafted from hard woods, including ebony (Figure 4b), metal, or ivory. By the late 19th and 20th centuries, chrome plating of surgical instruments became universal to prevent corrosion, as seen in knives from the 1920s (Figure 4c). Today, these tools are manufactured from surgical stainless steel and other metals like titanium, while modern tissue cutting also utilizes lasers or ultrasound. In standard hospital conditions, however, the basic attributes remain the steel amputation knife (for soft tissues) and the manual bone saw (for bone), as the saw avoids tissue crushing and minimizes bone splinters compared to axes or chisels commonly used in Roman times [46] (Figure 4d,e).

**Figure 5:**
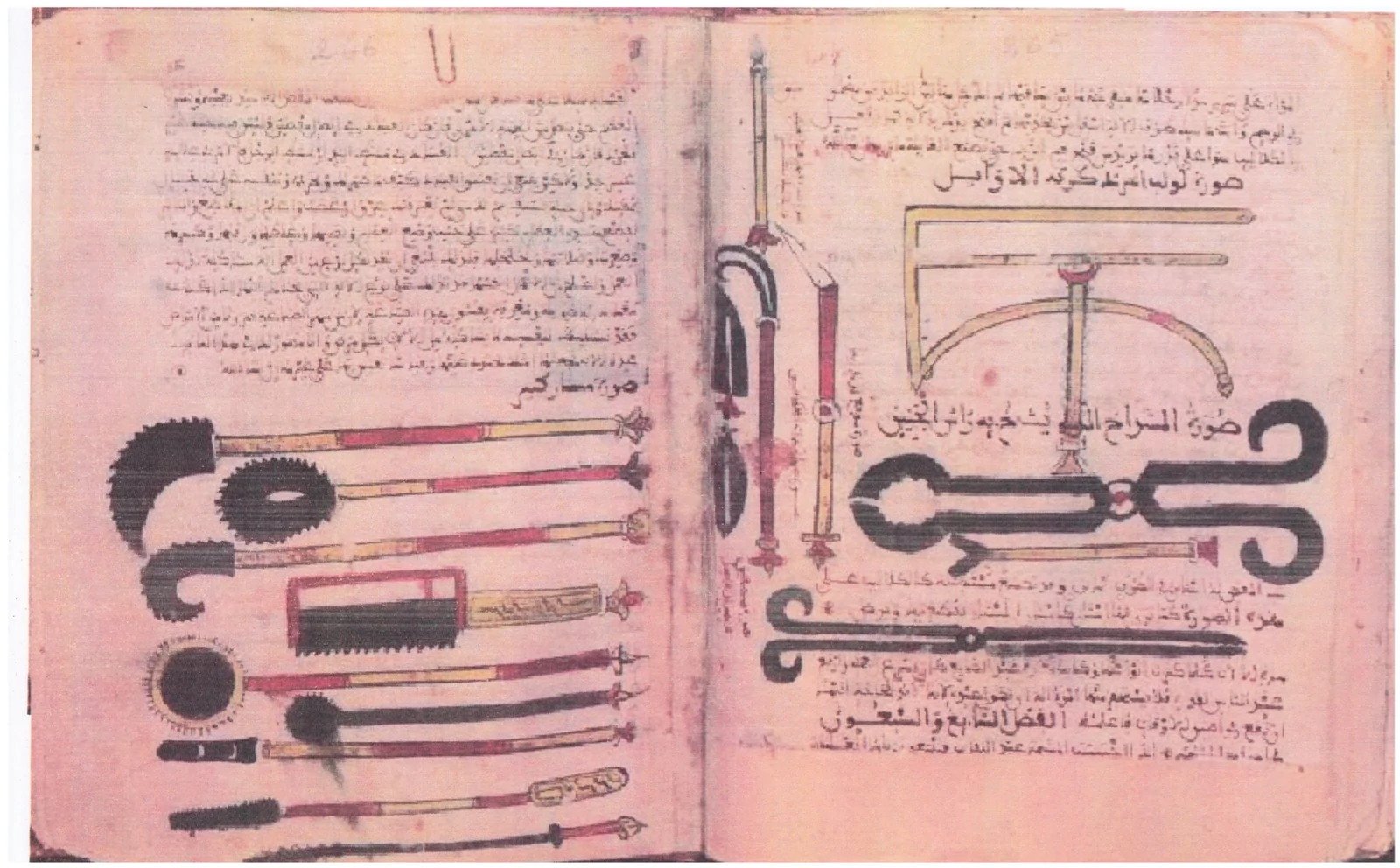
Types of amputation saws shown in the book of Abulcasis „*Kitab Al.-Tasrif*” (The method of medicine) From: Mc Gill Libraries Bibliotheques; Islamic Manuscripts, Art collection and Rare Books at Walters Art Museum; Ghazaleh Ghanavizchian (online); [https://blogs.library.mcgill.ca/islamicstudieslibrary/files/2023/09/image-4.png (accessed on 9 August 2026), Public Domain] [50].

A medieval surgeon performing an amputation was typically equipped with a knife, a saw, and the needles, threads, or forceps needed to dress the wound [49]. A large array of ancient surgical tools, including various types of amputation saws, was presented in Abulcasis’s “*Kitab Al-Tasrif*” (Figure 5). Medieval Islamic veterinary surgery in parallel developed its own specialized iron instrumentarium; Mamluk treatises illustrate the use of specialized curved and straight cauterizing tools, forceps for removing parasites from head structures, and restraint apparatuses made of iron, wood, leather, and ropes to manage locomotor disorders [10].

**Figure 6:**
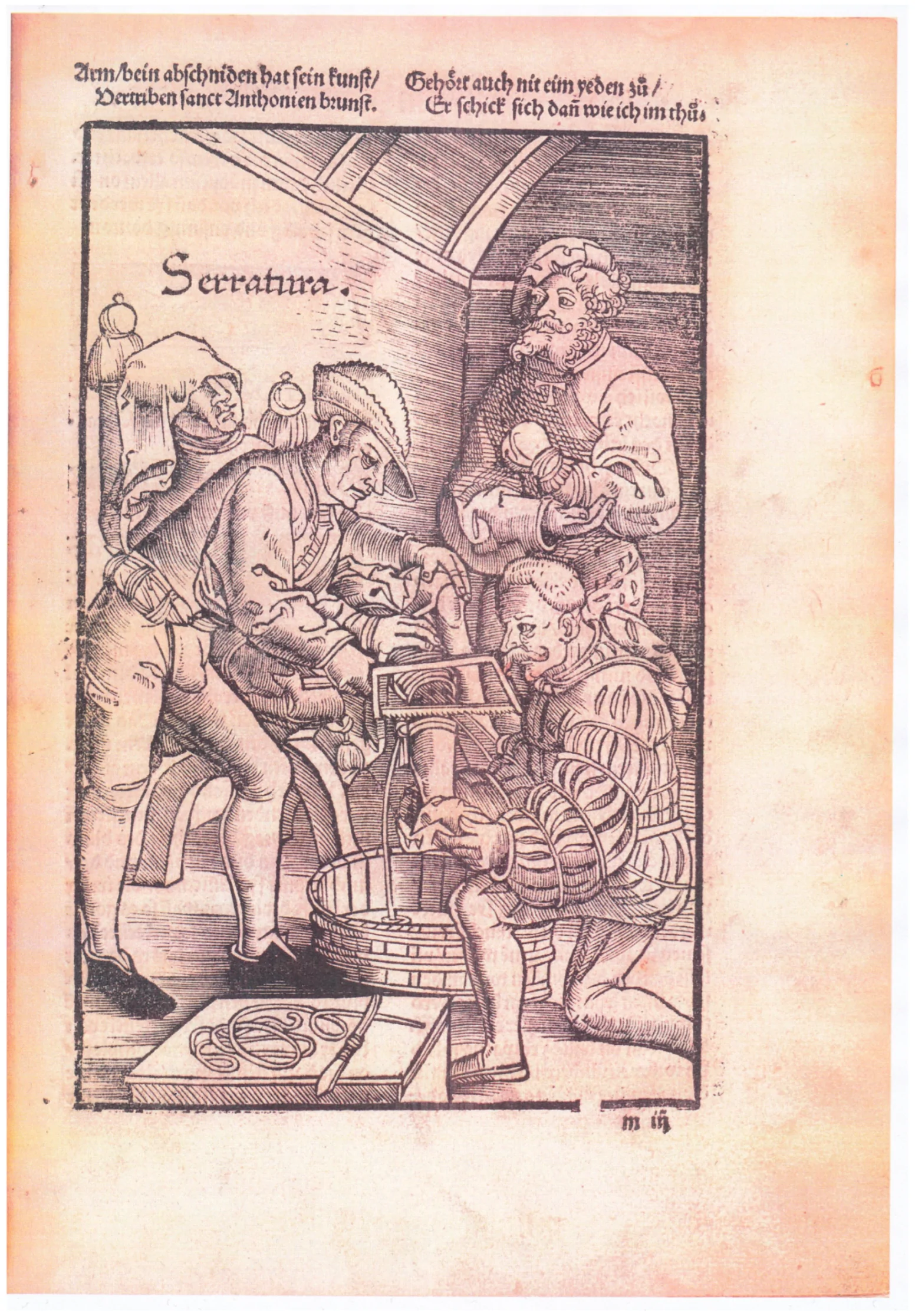
Amputation—figure from the book: Hans von Gersdorf „*Feldbuch der Wundartzney*” (Field book of surgery), Strassburg, J. Schott, 1517. From: auction catalog Bonhams (online); [https://www.bonhams.com/auction/29491/lot/51/gersdorff-hans-von-1455-1529-feldtbuch-der-wundartzney-strassburg-j-schott-1517/ (accessed on 9 August 2026)] [52].

**Figure 7:**
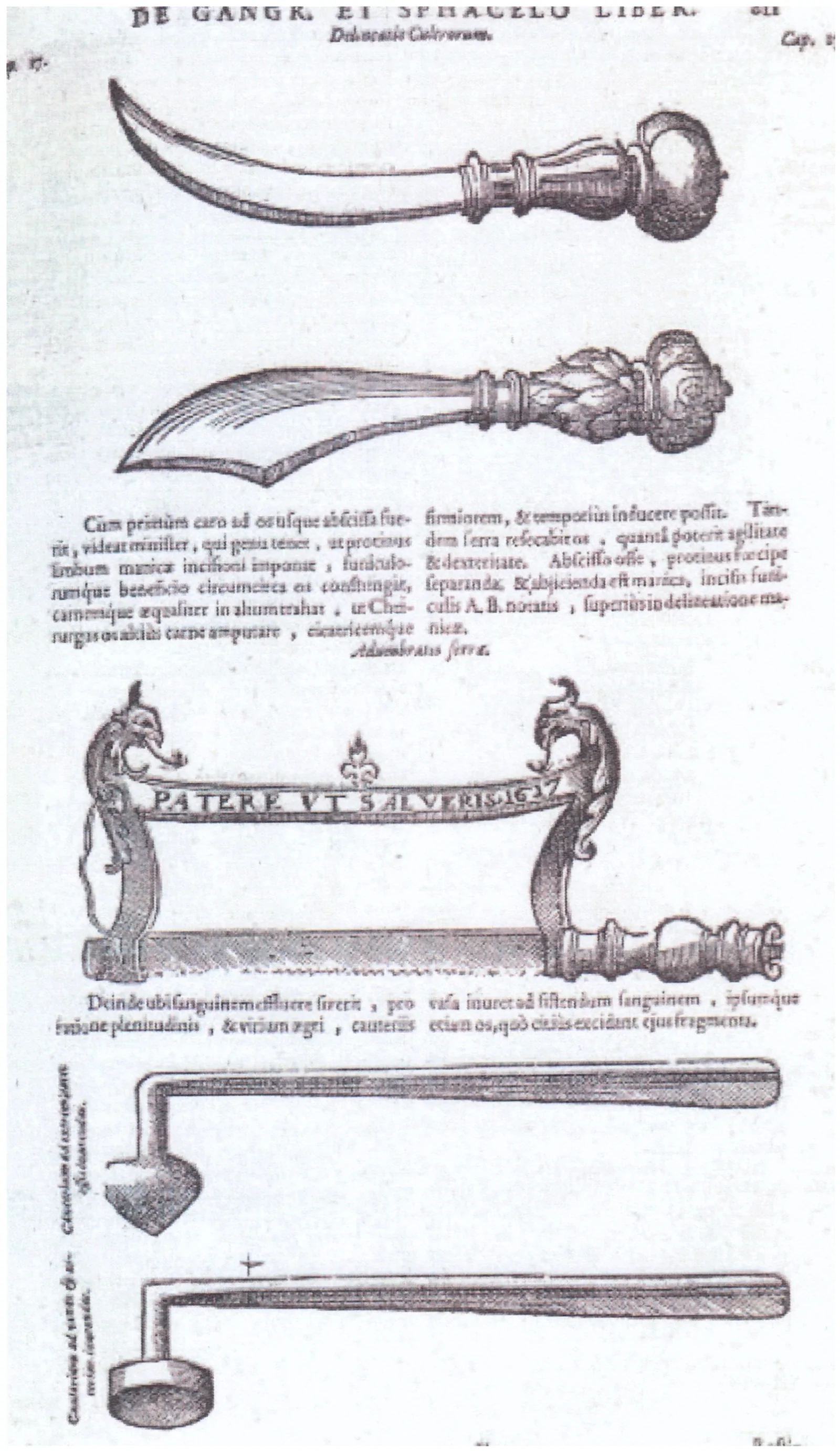
Amputation knives, amputation saw and tool for cauterization—figure from the book: Fabricii Hildani G., „*Opera Obervationum et Curationum Medico-Chirurgicarum*” (Collected Works on Medical and Surgical Observations and Treatments), Frankfurt 1682 [51].

**Figure 8:**
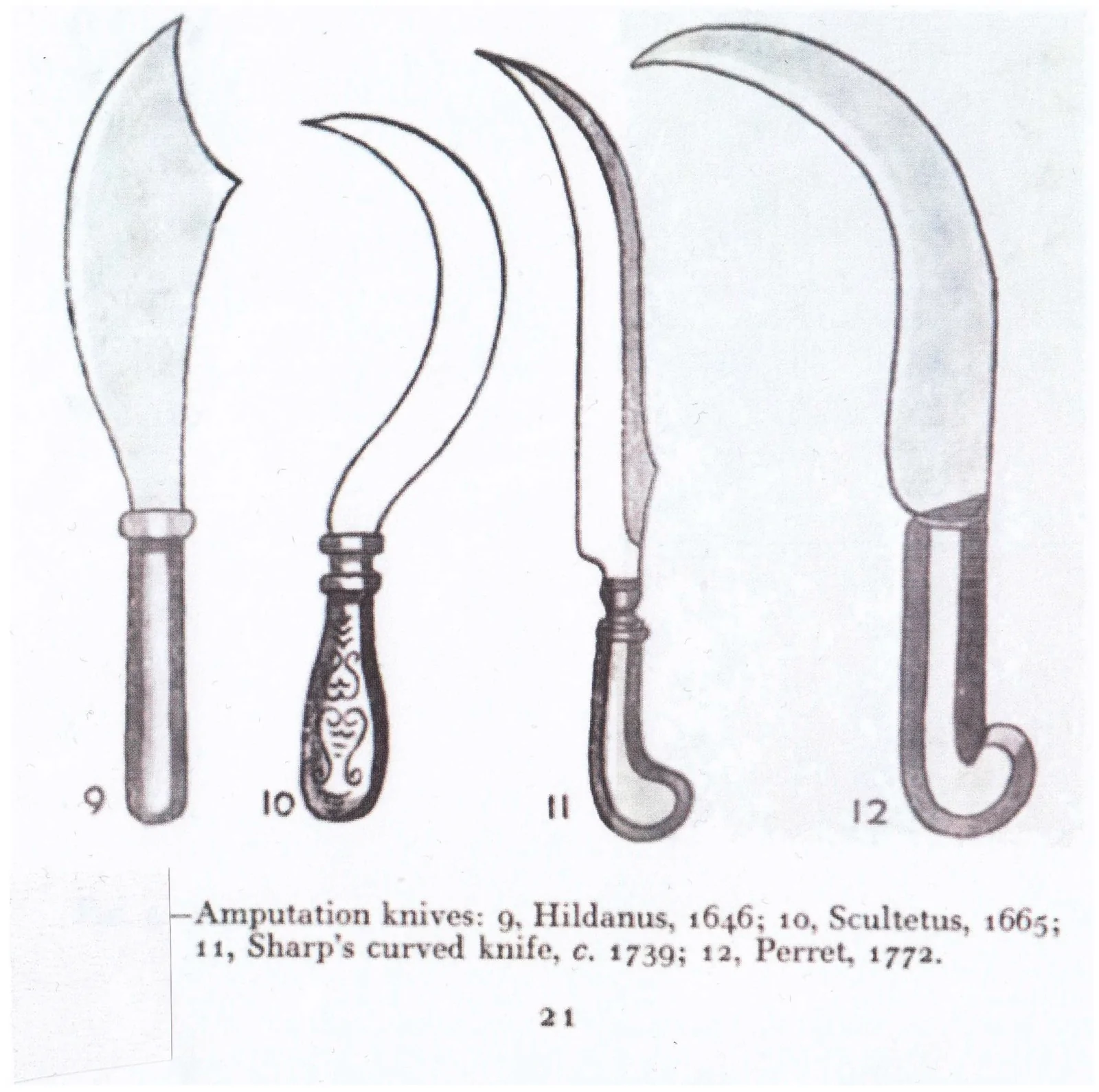
Amputation knives, figure from the book: Thompson, C. “*The History and Evolution of Surgical Instruments*” (1942) [53]. From: https://oldoperatingtheatre.com (accessed on 9 August 2026), Public Domain.

**Figure 9:**
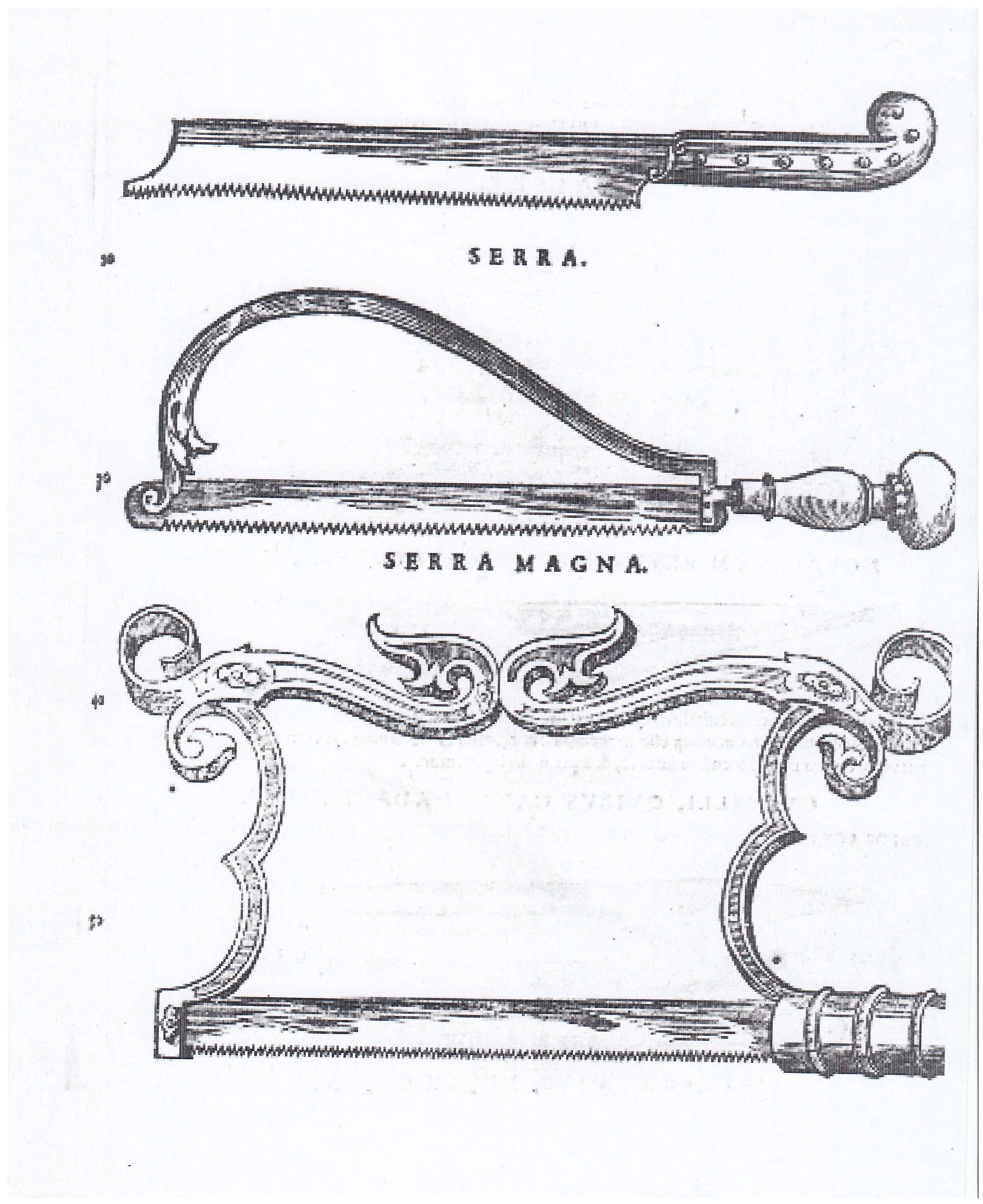
Amputation saws, figure from the book: Croce JA, „*Chirurgiae Libri Septem*” (Seven Books on Surgery), Venetiae, G. Zilleti, 1573. From: Kirukup J., „A history of limb amputation”, Springer—Verlad London 2007 [32].

In subsequent centuries, this technique was continuously visualized: a physician sawing a patient’s lower leg bone with a hand saw was depicted in Hans von Gersdorff’s “*Feldbuch der Wundartzney*” from 1517 [27] (Figure 6), and knives, a bone saw with the Latin inscription *patere ut salveres* (“suffer to be saved”), and instruments for wound cauterization were shown in Wilhelm Fabry’s published work [27,51] (Figure 7). Surgical knives across different eras featured single and double-sided blades, straight or curved configurations [33,34,46] (Figure 8), while amputation saws were most commonly straight, varying in handle shape and blade mounting methods [32,33] (Figure 9).

## 5. Surgical Techniques and Operative Approaches

Amputation techniques can be fundamentally divided into joint disarticulation and amputation with bone transection. Relatively little is known about the earliest methods. The oldest known medical amputation from 31,000 years ago in Borneo involved sawing the lower leg bones, whereas the 7000-year-old amputation from France involved removing the limb at the elbow joint [2,3]. Hippocrates advised incisions strictly within necrotic tissue below the “black line” of discoloration to avoid causing pain or severe haemorrhage in living tissues [28].

The Roman author Aurelius Celsus recommended amputation within healthy tissue to completely eradicate gangrene, providing methods for managing haemorrhage and advising against incisions near joints, though he treated the procedure as a last resort [54]. Celsus described that between the healthy and diseased part, the flesh is cut down to the bone, the healthy flesh is retracted and cut around the bone to expose it, allowing it to be sawed off with a small saw as close to the healthy flesh as possible so that sufficient skin remains to cover the bone [54]. This circular incision method remained dominant until the 19th century [54]. A two-stage circular cut—cutting the skin below the subsequent cut of muscle and bone—was introduced by Jean Louis Petit (1674–1750) [27].

**Figure 10:**
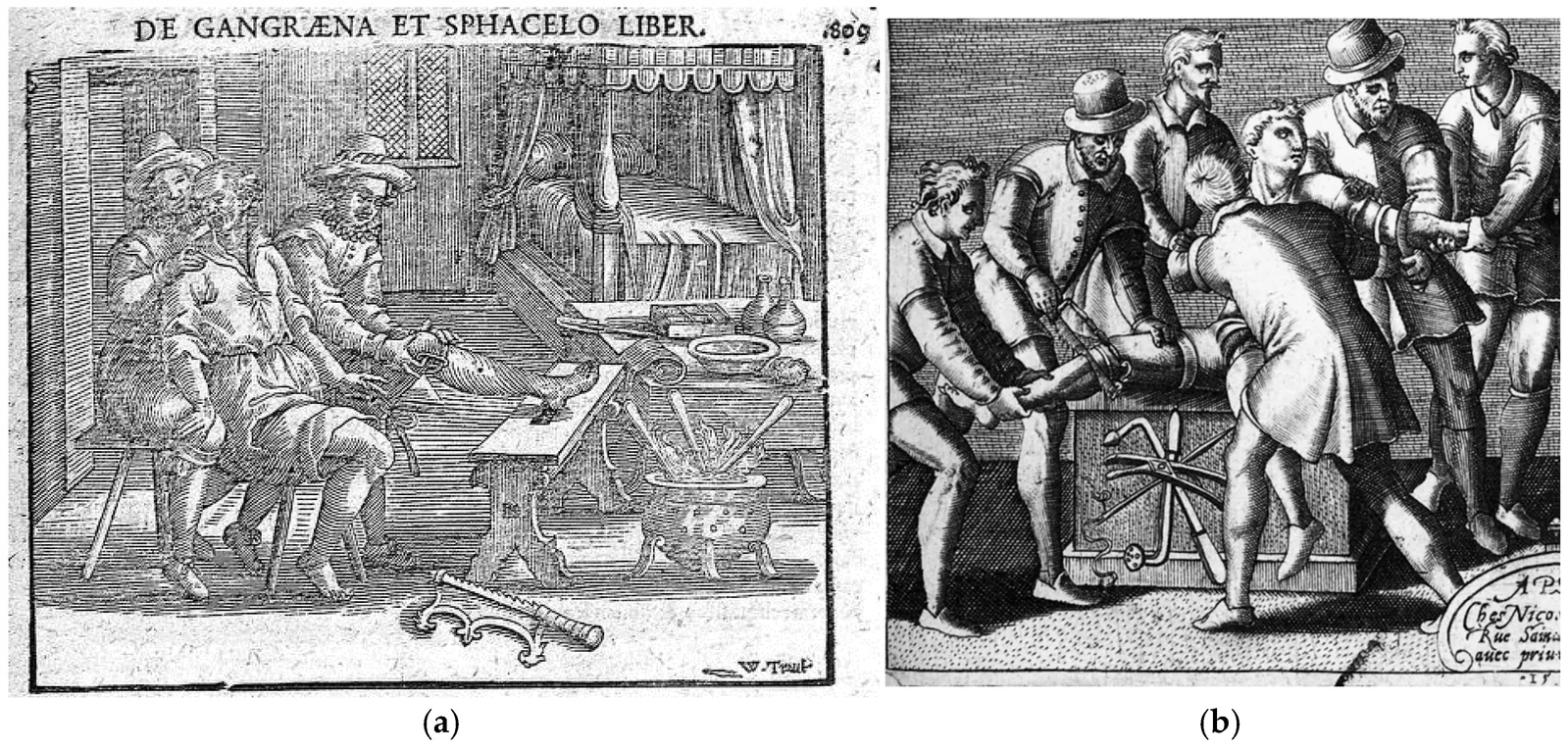
Amputations; Various amputation tools also visible in both engravings. (**a**): Figure from Fabricii Hildani G., „*Opera Obervationum et Curationum Medico-Chirurgicarum, Liber de Gangrena et Sphacelo*” (Collected Works on Medical and Surgical Observations and Treatments, book ”On Gangrene and Sphacelus”), Frankfurt 1682 [51]; (**b**): Figure from Guillemeau J. “*La Chirurgie Francoise”* (French Surgery), 1594—fragment of the front page. Fom: Kirukup J., „*A history of limb amputation*”, Springer—Verlad London 2007 [32].

**Figure 11:**
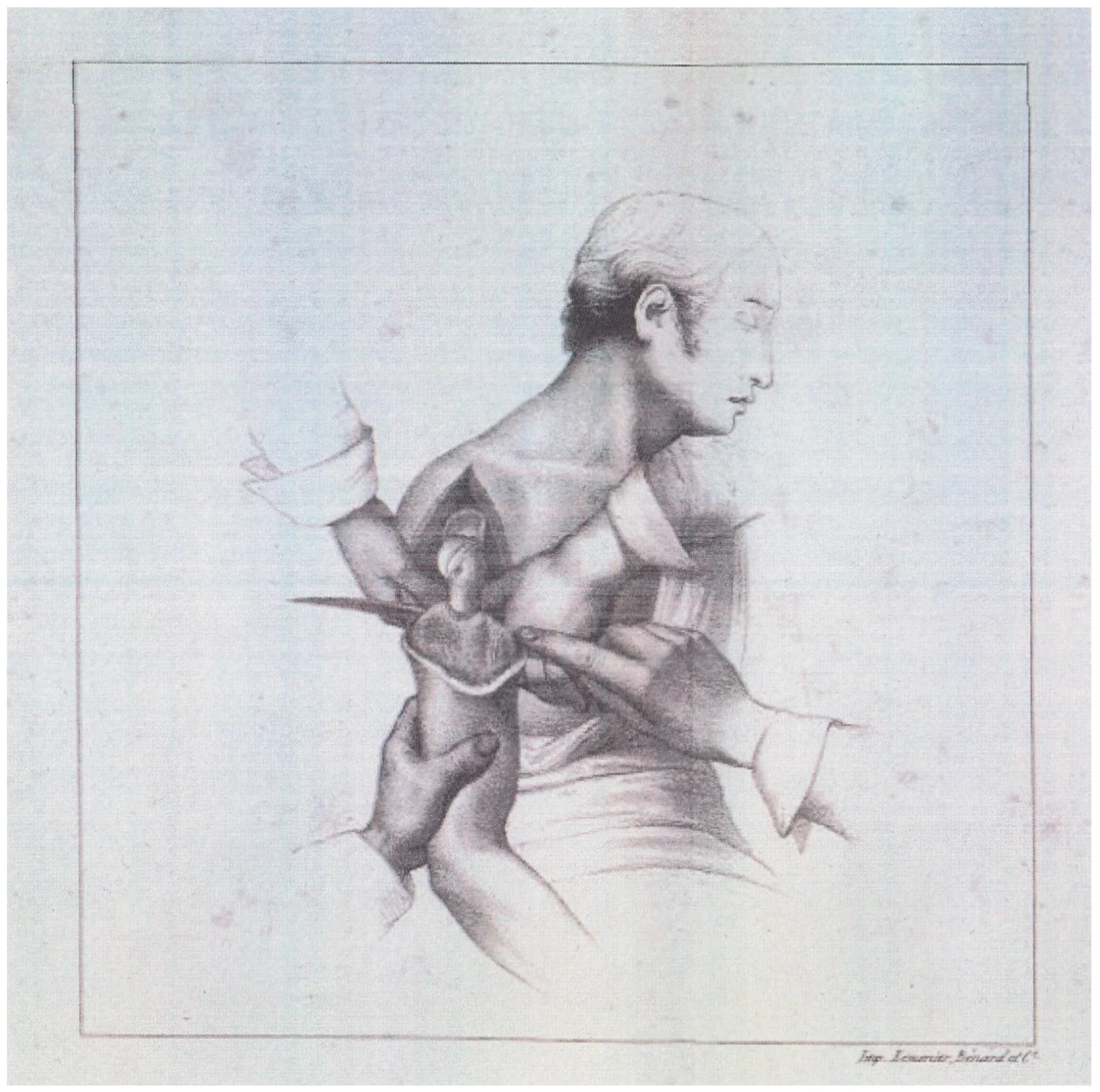
Amputation by the shoulder joint disarticulation. Drawing by Jean Dominique Larrey, “*Memoires*” around 1816 (in other editions known, among others, as “*Memories of a military surgeon*”). From: “*Amputations of limbs during the Napoleonic wars*” (online) [http://napoleon.org.pl/index.php/artykuly-historyczne/historia-medycyny/247-amputacje-konczyn-w-okresie-wojen-napoleonskich (accessed on 11 June 2025)] [59].

Guy de Chauliac described both disarticulations and transosseous amputations in “*Chirurgia Magna*” (1367) [32]. These procedures were most frequently performed in a seated position with the patient restrained by assistants (Figure 6 and Figure 10a,b). Joint disarticulations became highly popular during the Napoleonic Wars, as detailed by Jean Dominique Larrey [55] (Figure 11). When a trend emerged to complete amputations in the shortest possible time to reduce patient suffering, this idea led to absurdities: historical accounts claim that a lower limb disarticulation at the hip joint could be performed in as little as 19 s, speed that practically precluded any meticulous surgical care [56]. This era of rapid, pre-anesthetic surgery is famously epitomized by the iconic historical anecdote of Robert Liston, who reportedly performed a thigh amputation in under three minutes with a purported 300% mortality rate due to the accidental maiming of his surgical assistant and a spectator. While modern historians frequently debate the precise statistical authenticity of this specific event, its prominence in medical history effectively illustrates the extreme risks, profound psychological pressures, and compounding complications that defined surgical speed before the advent of chemical anesthesia [57]. Today, human lower limb amputations prioritize the residual stump’s capacity for prosthetics, ensuring it can withstand pressure and maintain an adequate range of motion [58].

In veterinary surgery, the operative technique in horses shows a close structural analogy to historical and modern human methods, relying on joint disarticulation or transosseous amputation [4]. Published case reports describe limb removal in the distal segment—most commonly approximately 10 cm distal to the tarsometatarsal joint (clinically corresponding to the human Lisfranc joint line) [4,60]. To minimize pain complications, performing pre-operative regional nerve blocks (peroneal and tibial nerves) using mepivacaine under general anaesthesia has been described in published equine case reports [60]. A critical post-operative element is the immediate application of a rigid synthetic fiberglass cast extending to the hock to prevent edema and stabilize the stump [60,61]. Weight-bearing requires complete primary intention healing; until then, the surgical tissue cannot directly accept body mass, necessitating specialized load-transferring solutions [20]. Biomechanical risks were recognized by Scaccho [13], who forbade deep fire cauterization for fresh fractures or dislocations, recommending manual reduction and rigid splinting instead.

In advanced orthopedic protocols for horses, stabilization is achieved via two centrally threaded positive-profile transfixation pins (6.3 mm diameter) inserted into the distal third of the metacarpal bone at a divergent 30° angle [62]. Divergent angles drastically reduce recovery fractures compared to traditional parallel pinning [63]. To offload soft tissues, a temporary aluminum cup apparatus is incorporated into the cast, providing a stable pivot point and reducing torque [57]. Although distal disarticulation via a caudal flap technique is preferred in horses, transosseous long-bone amputations are performed using a “fish-mouth” incision, where each skin flap length corresponds to 3/4 of the limb diameter [64].

Similar dilemmas apply to oncological amputations in small equids [65]. In a Shetland pony mare treated for squamous cell carcinoma of the hoof laminae, an initial digital disarticulation at the metacarpophalangeal joint via a palmar flap technique required surgical revision to a higher transosseous amputation at the proximal shaft of the third metacarpal bone [65]. Although historical attempts in 1922 failed due to rapid recurrence, a higher amputation achieved a two-year recurrence-free recovery [66]. The prohibitive biomechanical challenges and complications observed in heavy horses contrast sharply with outcomes in lighter species, where a lower body mass allows for more radical bioengineering innovations. Consequently, modern veterinary surgery successfully implements advanced techniques from human medicine in smaller ungulates, exemplified by a successful procedure in a four-year-old Arabian tahr (*Arabitragus jayakari*). Following a distal tibial amputation, a permanent intraosseous transcutaneous amputation prosthesis (ITAP) integrated directly with the bone tissue [67]. Analogous principles apply to camelids, where young alpacas (*cria*) undergo radiocarpal disarticulation using two U-shaped skin flaps [68]. Selecting a joint-level resection deliberately eliminates osteotomy, minimizing blood loss [68]. Crucially, this source report also evaluates the long-term outcomes of limb salvage in two adult female alpacas subjected to mid-tibia amputations due to severe osteomyelitis. Both adult patients adapted rapidly to a permanent prosthesis secured via a full-body harness; one mare survived for 4 years before succumbing to an unrelated *Salmonella* infection, while the second successfully lived with the device for 8 years, with both individuals successfully breeding and giving birth to healthy offspring without complications [68]. Young individuals display greater adaptive capacities than adults [69]. Similar clinical success with juvenile patients is observable in livestock medicine, where an amputation of the thoracic limb was documented in a one-month-old calf following severe dystocia, utilizing early prosthetic management [21]. Guidance on human amputation levels and general surgical margins during limb salvage serves as an introductory baseline for these animal interventions [70], while specific plastic techniques for managing the residual limb shape must be strictly followed to avoid direct pressure zones [4,61]. While high resections in calves yield an average survival of 2–3 years provided suture lines avoid direct pressure zones, digit amputations in cattle remain highly routine procedures with excellent prognoses [71]. Generally, smaller individuals like goats, sheep, or camelids represent superior candidates for high resections due to lower mass [69,72]. However, a critical post-operative phase occurs around two months after fitting orthotics, when complications such as wound infections or stump pressure ulcers typically manifest [72]. For soft-tissue remodeling, historical Polish veterinary medicine also widely implemented setons and apertures to induce controlled inflammation via irritating sub-cutaneous strips to stimulate the immune system [15].

## 6. Methods of Haemostasis and Historical Cauterization

Managing haemorrhage is a paramount concern in surgical amputations. The oldest universal method used until modern times was cauterization, using high temperatures to denature tissues, seal blood vessels, and sterilize the wound. However, it was highly traumatic, painful, and induced localized tissue necrosis and subsequent inflammation. The observation that gunshot wounds treated with boiling oil healed poorly compared to wounds dressed simply led Ambroise Paré to rediscover vascular ligation [34,73]. Celsus also recommended cauterization only as a last resort due to its trauma [54]. Nonetheless, lacking advanced knowledge, it remained a fast, accessible, and effective method for centuries.

**Figure 12:**
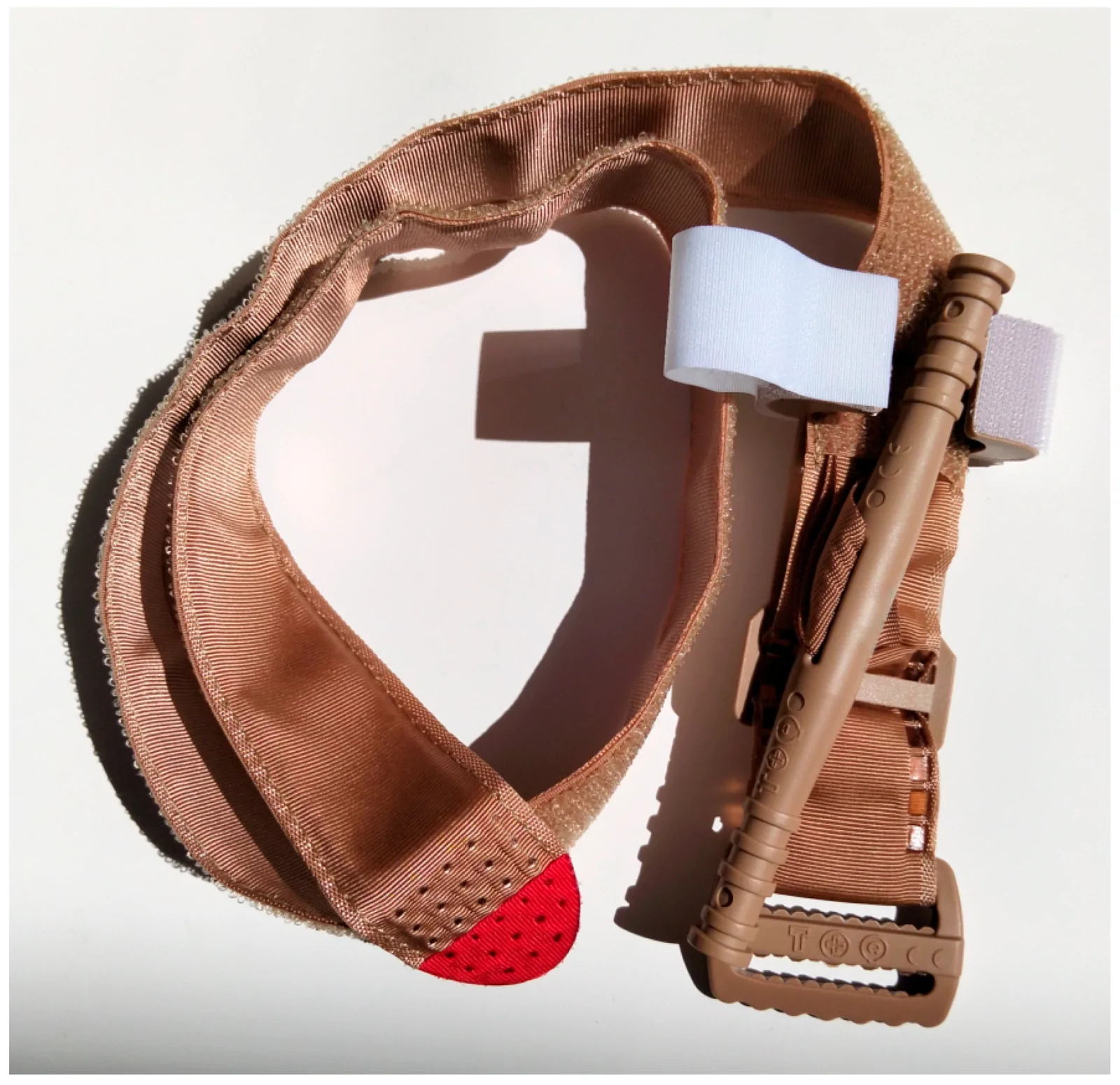
Combat Application Tourniquet (photo E.Pasicka, private collection, reproduced with permission).

Cauterization was performed via direct flames or boiling oil, though medical texts primarily advocated red-hot iron tools, consisting of metal plates attached to long handles [30,33,46,51] (Figure 7 and Figure 10b). Other historic haemostatic methods included wound packing (tamponade), pouring vinegar or wine, applying tight tourniquets (including the 1718 mechanical screw tourniquet), limb elevation, cooling, or applying astringents containing alum, vitriol, or turpentine [34,54,73]. Vascular ligation remains the most effective method. Ancient Greeks (including Hippocrates) did not practice ligation and avoided amputations out of fear of haemorrhage [34]. Hippocrates described only tourniquet compression applied to the limb rather than directly to individual vessels [73]. Vascular ligation was practiced by the Alexandrian school and described by Abulcasis, but the art was entirely lost in medieval Europe until Paré reintroduced it after 1545 [34,54,73,74]. Ligation was sometimes replaced by simpler tourniquets or screw tourniquets [27,32,51,55]. The fact that this knowledge was not universally applied stems from amputations being frequently performed by barbers and surgeons rather than educated physicians [75]. Today, human surgery utilizes ligation, injection methods, thermal electrocautery, and emergency field tourniquets [58] (Figure 12).

**Figure 13:**
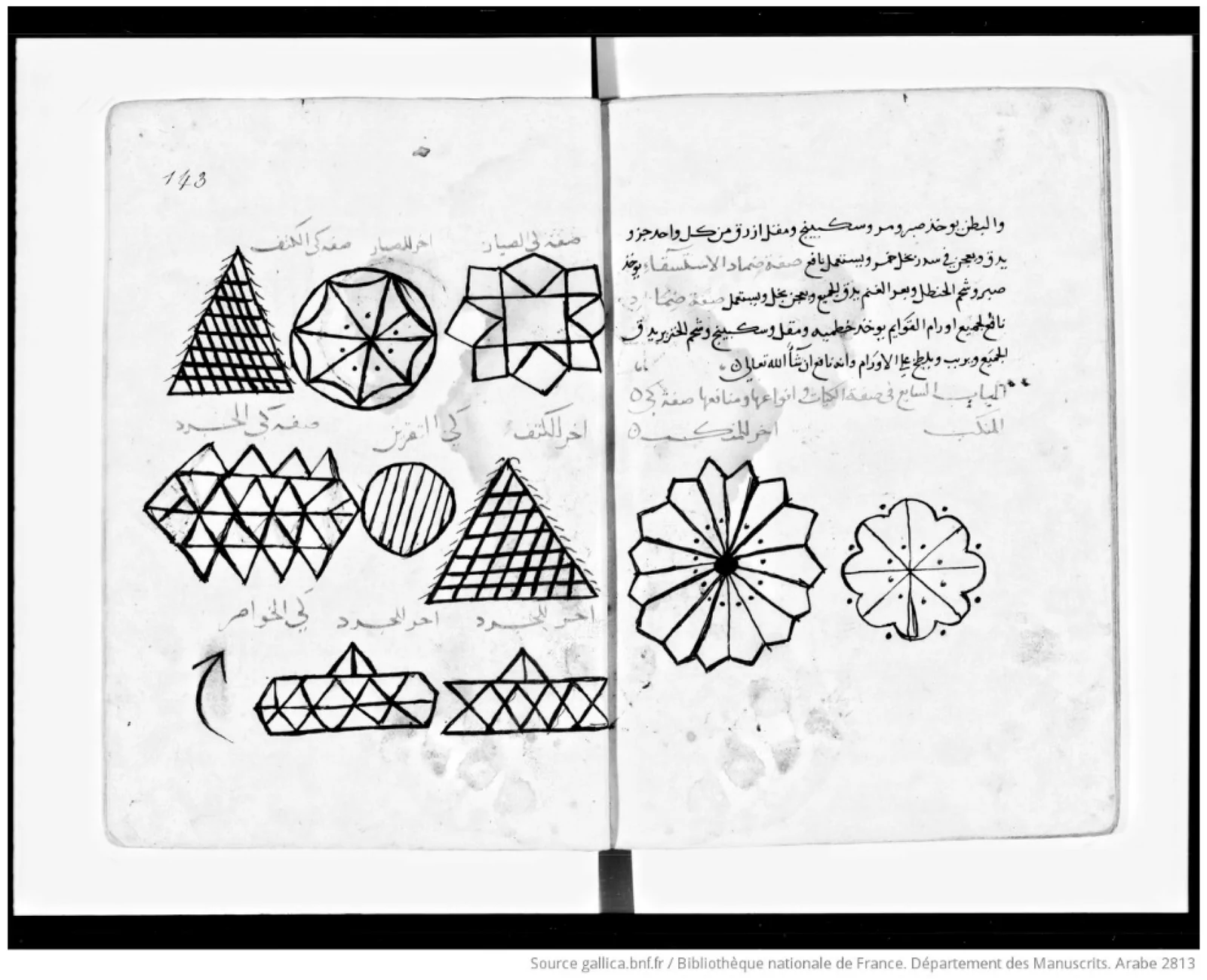
Folio 143v of the Mamluk manuscript “*Kāšif al-wayl fī maʿrifat amrāḍ al-ḫayl*” (The Revealer of Distress in the Knowledge of Horse Diseases). The polyhedral grids (**left**) represent geometric templates for symmetrical equine cauterization, while the text and circular charts (**right**) detail the pharmacological management of limb tumors and the systematic application of thermal surgery (*al-kayyāt*). Source: National Library of France (Bibliothèque nationale de France), MS Arabe 2813, 1471; Public Domain [76].

In Mamluk traditions, thermal cauterization with red-hot iron constituted a fundamental haemostatic and sterilizing solution [10]. Veterinary textbooks from that era systematized geometric patterns of burns applied to blood vessels, hooves, and skin to manage haemorrhage [10]. This geometric approach is illustrated in the Mamluk treatise “*Kāšif al-wayl fī maʿrifat amrāḍ al-ḫayl*” (1471), where polyhedral nets (Figure 13, left) served as templates for applying symmetrical burns to equine joints. Concurrently, the text and circular charts (Figure 13, right) outline the pharmacological treatment of limb swellings and determine the exact timing and anatomical coordinates for safe cauterization (*al-kayyāt*) [76]. This reliance on thermal medicine was equally mirrored in early modern Europe, where alternative haemostatic methods were virtually non-existent. In Italy, Scaccho [13] systematized this technique. Similarly, fire and cauterization irons were considered an absolute surgical panacea in old Polish veterinary medicine [15]. It was only at the turn of the 19th and 20th centuries that traditional thermal cauterization was successively displaced by chemical cauterization based on concentrated lysol, silver nitrate, or mercuric chloride [15].

## 7. Anaesthesia

Amputations are exceedingly painful and traumatic. Ancient Greek and Roman medical authors recognised that severe pain could exert profound physiological effects and, in extreme cases, could itself lead to death [28,77]. Anaesthesia using natural agents like alcohol and narcotic herbal extracts was employed by primitive peoples, Sumerians, Assyrians, Babylonians, Persians, and Hindus [77,78]. It was used by the Alexandrian school and the Chinese physician Hua T’o, who prescribed cannabis extracts as an intoxicating agent [74]. Greeks, Romans, and Arabs (Avicenna) applied mandragora root extracts to painful wounds, though its systematic use in amputations remains unverified [78]. Paracelsus used laudanum (opium tincture) [77,79]. Despite knowledge of these substances, data indicating their systematic prescription by physicians as a standard procedure are often lacking, and consumption frequently depended on the patient’s own fear of pain [77,79]. Widespread human amputations were performed without any anaesthesia in Europe until the mid-19th century [34,55,77,79]. A turning point was the introduction of ether anaesthesia (1842) and chloroform (1847). Modern human amputations utilize general, regional, or local anaesthesia [58,80].

Due to the total lack of anaesthesia in 16th-century veterinary medicine, the prevention of haemorrhage and painful shock was rooted in astrology. Scaccho [13] mandated that operations and bloodletting be performed exclusively during a waning moon, avoiding specific organs when their corresponding zodiac sign dominated. Modern veterinary medicine utilizes general inhalation anaesthesia combined with advanced regional nerve blocks.

## 8. Prosthetics and Long-Term Cross-Species Complications

**Figure 14:**
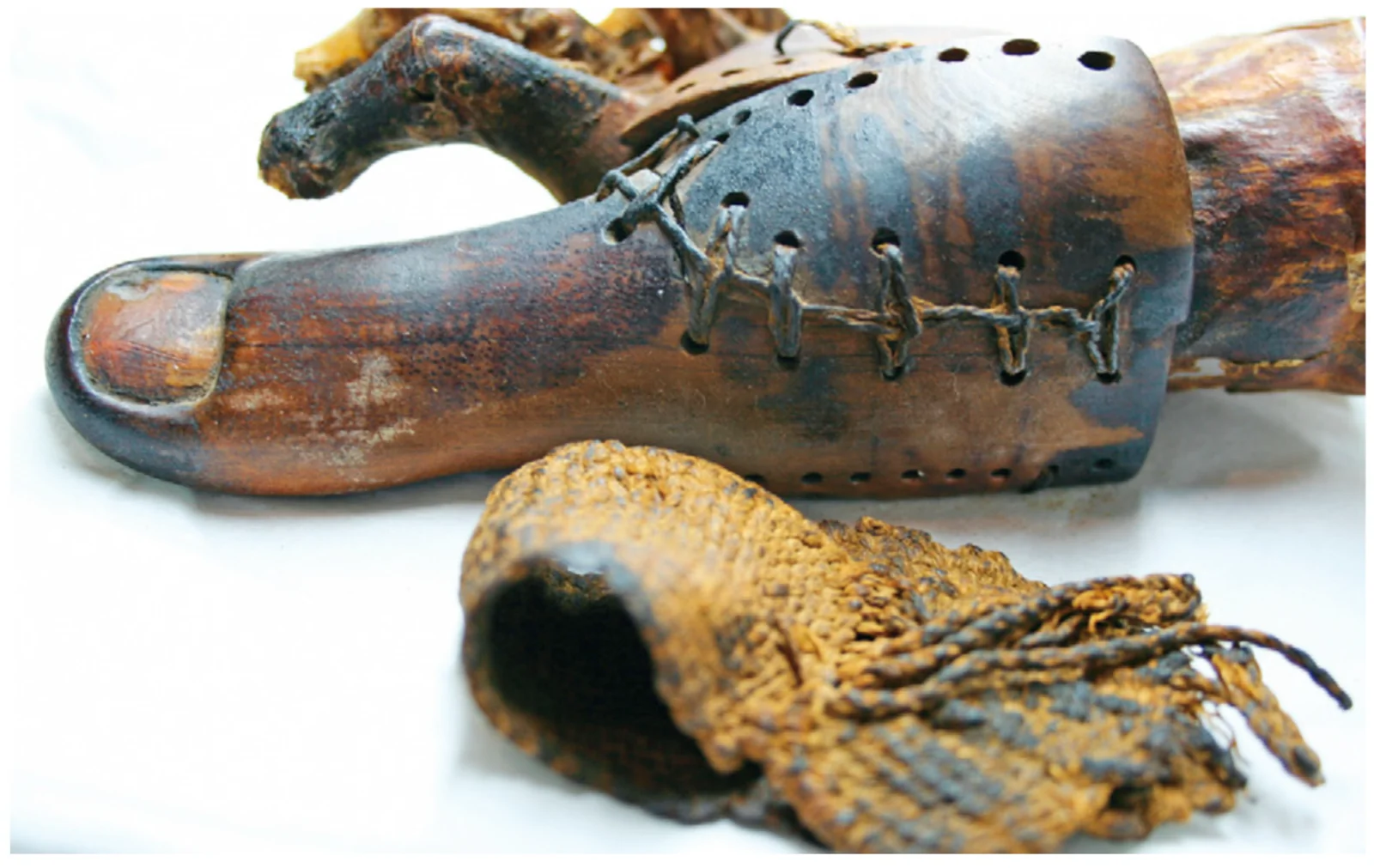
The prosthesis of the toe in the Egyptian mummy of a young woman buried near Luxor. From: Finch J., The ancient origins of prosthetic medicine, The Lancet, Volume 377, Issue 9765, 548–549 [81].

**Figure 15:**
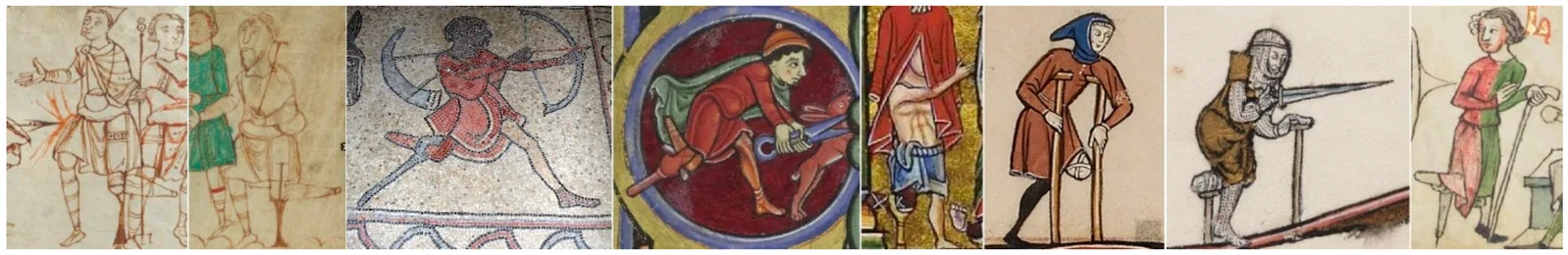
People with various types of prostheses shown in drawings and mosaics from 1201–1375. From: Vlasaty T., “Amputation of the Lower Limbs of the Early Middle Ages and Their Prostheses”, 2003 (online) [https://sagy.vikingove.cz/en/amputation-of-the-lower-limbs-of-the-early-middle-ages-and-their-prostheses] (accessed on 12 October 2026) [83].

Following limb loss, prosthetics developed to fulfill a dual purpose: improving patient mobility and mitigating visible deformity (*cosmesis*). Prosthetics often arose independently and earlier than medical amputation techniques to manage traumatic amputees. Archaeological records contain numerous ancient Egyptian prostheses, such as a functional prosthetic toe discovered on a young woman’s mummy buried near Luxor [27,34,81] (Figure 14). One of the earliest accounts of prosthetics is recorded in the ancient Indian *Vedas*, describing an iron limb made for the warrior queen Vishpala [24,82]. Historic human prostheses are known from drawings and mosaics from 1201–1375, depicting patients using devices resembling simple wooden pegs (Figure 15) [83]. Over centuries, wooden and metal designs were attached via various straps, whereas modern human prosthetics utilizes carbon fiber, aluminum, and titanium. Long-term assessment frameworks evaluating how human patients report subjective quality of life and issues of importance regarding their devices provide a conceptual methodology for animal orthotics [84]. Clinical studies tracking structural alterations in the physical interface over time demonstrate how the residual limb shape adapts to prolonged weight-bearing, reinforcing the need for flexible sockets [85]. Modern post-amputation protocols for these human subjects emphasize comprehensive occupational therapy and targeted exercises to safely maximize post-operative mobility [86,87].

Modern veterinary prosthetics heavily adapts these human concepts, utilizing socket-type cylindrical prostheses mounted on the long residual stump of the metacarpus or metatarsus [4]. In the forelimb, the socket is stabilized by the prominent carpal joint, whereas in the hindlimb, the angle above the hock provides primary support [4,23,88]. Alternatively, interlocking intramedullary devices are used [4,89]. Regardless of the method, the prosthesis socket must exhibit exceptional flexibility and residual elasticity [20]. Historic equine apparatus evolution began in the 1970s with duralumin designs featuring swinging inspection doors to facilitate hygiene and the application of drying agents like calcium oxide and scarlet oils [88].

An annoying complication masked by the socket is intense stump pruritus, managed via manual massage after apparatus removal [88]. Contemporary veterinary breakthroughs include the implementation of 3D-printing technology for growing camelids [68]. The modular construction of a 3D-printed prosthesis (flexible foot, shaft, and socket) allows for the sequential replacement of individual components in growing animals, radically lowering maintenance costs [68].

A shared pathomechanism of complications is observed between human and veterinary patients. Fluctuations in stump diameter over time alter internal friction points in horses, leading to chronic pressure sores, serous discharge, and recurrent bacterial infections manifesting as abscesses [4]. Chronic mechanical compression almost inevitably induces extensive pressure necrosis of the residual soft tissues [20]. The most critical and uniquely devastating complication in heavy veterinary patients—entirely distinct from human medicine—is the massive redistribution of load onto the contralateral (healthy) limb. The primary complication following such procedures is the catastrophic breakdown of the suspensory apparatus in this contralateral, weight-bearing limb. Due to prolonged, asymmetric static overloading, the suspensory ligament and its distal branches undergo severe degenerative changes, leading to a permanent drop of the fetlock joint, as well as secondary, often fatal, laminitis, representing independent manifestations of supporting-limb overload [4,23]. To protect against neuroma formation and phantom pain—which horses experience similarly to humans—surgeons implement free frog tissue grafts onto the stump [62].

Ultimately, success depends on strict candidate selection: the patient must exhibit an appropriate temperament to tolerate the loss of hydrostatic support, suspension in a specialized sling, and long-term medical manipulations [4,60]. It also demands substantial financial and time commitments from owners, as care is permanent [4]. The primary limitation in horses remains the extremely high rate of complications, illustrated by a 20-year-old Arabian stallion in Israel; despite initial surgical success performed 10 cm distal to the tarsometatarsal joint complex—anatomically homologous to the human Lisfranc joint line—long-term biomechanical and patomechanic changes induced progressive muscle atrophy in the contralateral hindlimb, forcing euthanasia two years post-surgery [4]. Retrospective analyses indicate that distal equine amputation yields a 6-month survival rate of approximately 64.7%, with a mean overall lifespan of 31 months [20,90]. Ideal candidates are patients with acute injuries before the onset of osteopenia or contralateral limb overloading [62]. Unlike heavy horses, smaller ungulates (Shetland ponies, donkeys) benefit from low mass and natural rusticity, minimizing contralateral overload risks [62,65,91]. This is confirmed by a donkey surviving in full comfort without a prosthesis for 96 months [62] and a sambar deer ambulating freely for 10 months post-amputation [91]. Excellent biomechanical adaptation is likewise noted in prosthetic-fitted alpacas, confirming their superior therapeutic suitability for limb salvage compared to heavy equids [68]. A comprehensive synthesis of these reported clinical cases, including variations in body mass, levels of resection, and distinct therapeutic outcomes across diverse animal species, is systematically structured in Table 2.

## 9. Conclusions and Future Directions

This comparative review indicates that the historical development of amputation in humans and ungulates followed partially interconnected but ultimately divergent trajectories. In response to the first research question regarding the transfer of surgical knowledge, advances initially developed or systematically documented in human surgery—including surgical instrumentation, vascular control, anaesthesia, operative techniques, and prosthetic concepts—provided important foundations for subsequent veterinary applications. However, this transfer was neither linear nor universal, as early modern veterinary medicine consistently resisted radical resections until 20th-century advancements in biomaterials.

Addressing the second research question, the distinct anatomical and biomechanical boundaries of large animals represent the principal limitation to long-term success. High body mass prevents direct immediate weight-bearing on the residual limb, forcing a massive redistribution of load to the contralateral healthy limb. This asymmetric static overloading may result in independent, severe complications, namely, musculoskeletal deterioration, suspensory apparatus failure, and secondary laminitis. While these conditions can be fatal in some cases, the available clinical evidence does not support the conclusion that fatal laminitis is an inevitable outcome. Reported outcomes vary considerably according to body mass and rehabilitation, meaning smaller ungulates offer significantly more favourable biomechanical conditions for partial amputation. This clinical dichotomy is strongly supported by the integrated zooarchaeological and paleopathological evidence. The profound taphonomic absence of fully remodelled amputation stumps in the large ungulate fossil record confirms that under historical husbandry conditions, severe long-bone fractures or irreversible distal limb trauma almost invariably resulted in immediate economic culling or rapid death from vascular necrosis and contralateral limb overload. While ancient populations could successfully manage non-displaced greenstick fractures or maintain lame equids through chronic ankylosing phases like bone spavin, true structural amputation remained an absolute biological boundary. This historical baseline underscores that the long-term survival of heavily disabled large quadrupeds could not be achieved through empirical folk treatments, but strictly required the technological paradigm shifts and advanced biomechanical support introduced by modern veterinary bioengineering.

Regarding the third research question, modern bioengineering and changing animal-welfare paradigms have successfully redefined the clinical status of these patients. Innovations like 3D printing and intraosseous transcutaneous amputation prostheses (ITAP) have expanded the range of potentially salvageable candidates. Simultaneously, the cultural transition from viewing animals through a purely utilitarian lens of livestock commodity to recognizing them as sentient companion beings has strengthened the medical motivation to pursue life-saving functional rehabilitation rather than routine economic culling.

## Figures and Tables

**Table 1:** Comparative Trajectories of Human and Veterinary Amputation across Key Historical Periods.

Historical Period	Human Medicine Trajectory and Outcomes	Veterinary Medicine Trajectory and Outcomes	Key Enabling Technologies	Primary Limiting Factors and Complications
Prehistory and Antiquity	Standard life-saving attempt for trauma/gangrene; long-term survival documented (e.g., Borneo, France).	Utilitarian culling or destructive military maiming (hamstringing); no therapeutic context.	Sharp stone tools; bronze/iron blades; early wooden prostheses (pegs, toes).	Complete lack of vascular control; massive surgical shock; lethal hemorrhage.
Islamic Golden Age and Middle Ages	Formalized surgical protocols (Abulcasis); standard clinical treatments for ischemic limb gangrene.	Emergence of advanced equine fracture management and splinting (Al-Baytar).	Specialized iron saws and knives; geometric thermal cauterization irons.	Severe soft-tissue ischemia; reliance on thermal destruction for hemostasis; no anesthesia.
Early Modern Era (16th–18th C.)	Reintroduction of vascular ligation (Paré); two-stage circular bone cuts (Petit).	Superficial hippiatry (Scaccho); bloodletting via fleams; complete avoidance of limb resection.	Mechanical screw tourniquet; specialized curved knives; vernacular treatises.	Lack of anatomical offloading capabilities; extreme procedural pain causing shock; fear of losing leg continuity.
19th to Mid-20th Century	Transition to rapid operating theater speeds (Liston) followed by the dawn of modern painless bone resection.	Amputation rejected for economic reasons due to rural mechanization; limited to saving high-value breeding mares.	Ether and chloroform anesthesia; antiseptic and aseptic techniques; chrome-plated tools.	High infection rates prior to Listerism; severe publication biases masking failures.
Contemporary Bioengineering	Prioritization of residual stump dynamics, flexible sockets, and high-performance mobility.	Amputation and prosthetic care established as a humane alternative to culling in selected candidates.	Intraosseous Transcutaneous Amputation Prostheses (ITAP); 3D printing; fiberglass casting.	Contralateral limb overload leading to secondary laminitis (heavy equids); stump pressure necrosis.

**Table 2:** Synthesis of Reported Clinical Cases, Biomechanical Constraints, and Therapeutic Outcomes.

Patient Species and Breed	Approximate Body Mass	Level of Limb Resection and Affected Limb	Orthotic/Prosthetic Strategy	Survival Duration	Primary Clinical Outcome, Complications, and Cause of Death	Primary Reference
Arabian-cross Stallion	330 kg	Right hindlimb (pelvic limb); 10 cm distal to the tarsometatarsal joint	Custom carbon-fiber socket prosthesis (initially temporary PVC tube)	24 months	Euthanasia due to the structural collapse of the contralateral (healthy) limb. Chronic complications included recurrent stump abscesses and friction-induced wounds due to changes in stump diameter.	Kelmer et al., [4]
Shetland Pony (Mare)	136 kg	Right forelimb (thoracic limb); amputated at metacarpophalangeal joint, then revised to 5 cm distal to the carpo-metacarpal joint	Custom socket-type prosthesis (thermoplastic polymer cup, stainless steel foot, gel-based stump cover)	26 months	Alive; successfully ambulating and able to go out daily in a paddock. Chronic small pressure sores continued to appear intermittently on the stump. Zero tumor (SCC) recurrence.	Moulin et al., [65]
Holstein Calf (Heifer)	Not stated (1-month-old calf)	Right third and fourth metacarpus (due to fracture), amputated at the radiocarpal joint (carpal disarticulation)	External aluminum and fiberglass shaft prosthesis with straps (capable of periodic lengthening for growth)	Approx. 2.5 years	Death due to reasons unrelated to the limb problem. Successfully entered the milking herd and produced a live calf. Suffered occasional stump abrasions and ulcerations.	Orsini et al., [21]
Arabian Tahr (*Arabitragus jayakari*)	15 kg	Left hindlimb (pelvic limb); distal aspect of the left tibia	Intraosseous Transcutaneous Amputation Prosthesis (ITAP) titanium implant + carbon fiber exoprosthesis	6 months	Alive; successfully reintroduced to the breeding group. Chronic complication: developed an Old-World screwworm fly larval infestation at the skin-implant junction (resolved surgically).	Golachowski et al., [67]
Alpaca (Male *cria*)	Not stated (16 days old at surgery)	Right forelimb (thoracic limb); radiocarpal joint disarticulation	Modular 3D-printed prosthesis (flexible foot, adjustable shaft, custom socket)	2 months (short-term follow-up)	Alive; comfortable ambulation in the herd. Developed a small pressure wound at the level of the stump after two months of use (treated successfully).	Vandermarcke et al., [68]
Alpaca (Female—case 1)	Not stated	Left or right hindlimb (pelvic limb); Mid-tibia	Permanent prosthesis attached to a harness/body sling	4 years	Death due to a *Salmonella* infection (unrelated to the prosthesis). Successfully gave birth to a cria. Experienced one pressure sore resolved by temporary removal of the device.	Vandermarcke et al., [68]
Alpaca (Female—case 2)	Not stated	Left hindlimb (pelvic limb); Mid-tibia	Permanent prosthesis attached to a harness/body sling	8 years	Alive; successfully living with the device and gave birth to two crias without complications. Requires maintenance/repair of the prosthesis 1–2 times a year.	Vandermarcke et al., [68]
Sambar Deer (*Cervus unicolor niger*)	Not stated (described as “young, adult”)	Right metacarpal fracture, amputated at the middle third of the humerus	None (pure tripedal adaptation; managed naturally on 3 limbs)	10 months	Alive; rehabilitated at a zoological park, confirmed to maintain good body condition and able to both walk and run.	Singh et al., [91]
Female Donkey (Nordestino breed)	95 kg	Left forelimb (thoracic limb); diaphyseal amputation through the midhumerus diaphysis	None (pure tripedal adaptation; managed naturally on 3 limbs)	8 years (96 months)	Alive; well adapted with good quality of life. Chronic complications included a stump fistula (healed in 60 days) and acquired deep digital tendon contracture on the right forelimb due to weight overload.	Câmara et al., [64]

## Data Availability

No new data were created or analyzed in this study. Data sharing is not applicable to this article.
